# A multi-compartment homogenized perfusion model for deforming hierarchical vasculature

**DOI:** 10.1007/s10237-025-02026-6

**Published:** 2025-12-24

**Authors:** Jannes Hohl, Adnan Ebrahem, Etienne Jessen, Marco F. P. ten Eikelder, Dominik Schillinger

**Affiliations:** Institute for Mechanics, Computational Mechanics Group, Technical University of Darmstadt, Darmstadt, 64287 Germany

**Keywords:** Tissue perfusion, Hierarchical vasculature, Synthetic tree generation, Multi-compartment homogenization, Darcy-type flow, Liver perfusion

## Abstract

The simulation of tissue perfusion based on highly detailed synthetic vasculature that often consists of multiple supplying and draining trees with millions of vascular segments is computationally expensive. Converting highly detailed synthetic vasculature into a homogenized continuum flow representation offers a computationally efficient alternative. In this paper, we investigate such a modeling approach that retains the essential features of potentially deforming hierarchical vascular networks. It is based on multi-compartment homogenization, where each compartment represents homogenized perfusion via a Darcy-type flow model associated with vascular segments at a specific spatial resolution in one individual tree of the network. The compartments are coupled through a pressure-dependent mass exchange, applied in a smeared manner everywhere within the perfusion domain. Key parameters, namely the permeability tensors of each compartment and the intercompartmental perfusion coefficients, are estimated directly from the vascular segments of the synthetic vasculature using averaging techniques. Our approach leverages spectral decomposition and a reduced set of representative vessel segments to balance computational efficiency with physical fidelity. For scenarios involving deformation, such as in a pumping heart or a regenerating liver, we introduce a computationally efficient parameter update based on geometric mapping, avoiding full re-homogenization in nonlinear simulations. We demonstrate the effectiveness and accuracy of the approach for several benchmark examples, including a full-scale multi-compartment liver perfusion simulation that explicitly incorporates three non-intersecting vascular trees, reflecting the hepatic artery, portal vein, and hepatic vein.

## Introduction

Over the last three decades, computational hemodynamics has advanced significantly, giving rise to diverse methods for modeling blood flow at various spatial scales. These methods range from detailed three-dimensional simulations of individual vessels to simplified one-dimensional network models and continuum-based homogenized representations (Formaggia et al. 2010; Caiazzo and Vignon-Clementel 2018).

Hierarchical vascular trees can be synthetically generated by optimization concepts, for instance built on Murray’s minimization principles (Murray 1926), and associated algorithms, such as constrained constructive optimization (CCO) (Schreiner and Buxbaum 1993; Karch et al. 1999; Schwen and Preusser 2012). However, traditional approaches are often limited to modeling only straight vessel segments and cannot efficiently handle large numbers of vessels. More recent synthesis methods overcome these constraints and have shown promising results for complex organs with high curvature, such as the human brain (Linninger et al. 2024). Vascular flow models with fully resolved network representations offer high fidelity, but present computational challenges due to the intricate geometry and scales of the vasculature. Homogenization techniques have emerged as a computationally efficient alternative, enabling the representation of microscale perfusion phenomena in a continuum framework. Darcy-type porous media models are frequently employed for this purpose, facilitating macroscopic descriptions of blood flow. Such models have been successfully applied to simulate microcirculatory flow in the heart (Chapelle et al. 2010; Richardson et al. 2021), lungs (Kowalczyk 1993; Patte et al. 2022), and the liver (Bonfiglio et al. 2010; Debbaut et al. 2014; Ricken et al. 2015; Rohan et al. 2018; Siddiqui et al. 2024; Lambers et al. 2024). Other approaches combine microcirculatory models with explicitly resolved vascular networks (Berger et al. 2016; Ebrahem et al. 2024; Debbaut et al. 2010; Stoter et al. 2017; Kremheller et al. 2019, 2021; Papamanolis et al. 2021; Menon et al. 2024; Sweeney et al. 2024; Cattaneo and Zunino 2014).

To capture the multi-scale nature of hierarchical vascular networks in a continuum approach, one can resort to multi-compartment homogenization, where the vasculature is partitioned into coexisting spatial compartments, each modeled using Darcy’s law (Rohan 2006; Cimrman and Rohan 2007; Cookson et al. 2012; Hyde et al. 2013, 2014; Shipley et al. 2020). These compartments are coupled via appropriate mass exchange terms, forming a multi-compartment perfusion model that can capture flow dynamics across multiple scales. This approach has been successfully applied to modeling perfusion in the liver (Ebrahem et al. 2025; Rohan et al. 2018) and in the heart (Cookson et al. 2012; Michler et al. 2013; Di Gregorio et al. 2021), where the latter has been extended to account for vessel compliance (Barnafi Wittwer et al. 2022; Montino Pelagi et al. 2024). A crucial aspect of the multi-compartment framework is the appropriate calibration of model parameters, such as compartment permeability and inter-compartment coupling, to accurately represent the underlying vasculature.

A general multi-compartment modeling framework for networks with tube-like vessel segments has been developed in Huyghe et al. (1989a, 1989b); Huyghe and van Campen (1995a, 1995b); Vankan et al. (1996, 1997, 1998), where a geometric formulation for permeability was derived. Additionally, a simplified mass exchange relation between compartments was proposed in Coussy (2004) and formulated explicitly in Hyde et al. (2013) for the application to such resolved networks.

In this work, we extend the multi-compartment Darcy-type perfusion model (Hyde et al. 2013; Michler et al. 2013; Hyde et al. 2014; Rohan et al. 2018) to deformable media, aiming for a unified framework compatible with poroelastic simulations. While the present study focuses exclusively on the perfusion aspect, we explicitly account for the possibility of tissue deformation by consistently distinguishing between the reference and current configurations. This is crucial because deformation alters vessel geometries, changing orientations and radii, and thus hydrodynamic resistance. Consequently, permeability tensors and inter-compartment coupling coefficients become deformation-dependent and require appropriate updating to reflect the evolving tissue state.

A primary contribution of this work is the development of an efficient parameter update strategy that avoids full re-homogenization in every iteration of a nonlinear large-deformation poroelastic simulation. Our approach combines spectral decomposition of the permeability tensor with re-homogenization performed over a substantially reduced set of representative vessel segments, thus balancing computational efficiency and model fidelity. Moreover, the proposed framework allows for integration of physiological assumptions–for example, we demonstrate an update strategy assuming constant hydrodynamic resistance in each vessel during deformation, motivated by applications in liver growth modeling.

A further contribution of this work is its specific application to liver modeling, driven by the organ’s complex vascular architecture. The liver contains three distinct blood-carrying vascular trees: the hepatic artery, portal vein, and hepatic vein. At the end of this paper, we present, to the best of our knowledge, the first multi-compartment perfusion simulation of the liver that explicitly includes three non-intersecting synthetic vascular trees. This enables a more realistic depiction of hepatic perfusion dynamics.

**Fig. 1:**
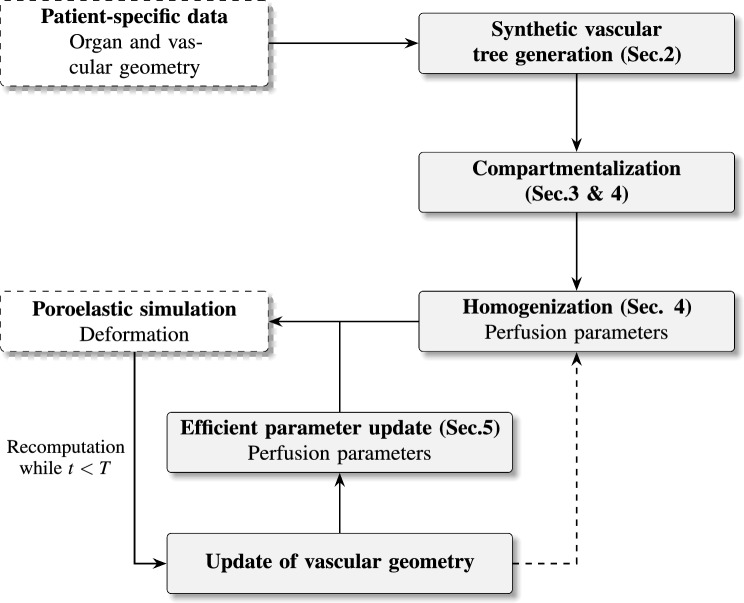
Flowchart of the modeling framework, indicating geometry generation, homogenization, poroelastic simulation, and parameter update steps. Elements not covered in the present work are shown in white with dashed outlines

An overview of the complete modeling framework is shown in Figure 1. Starting from imaging-based patient data, a synthetic vascular geometry is generated, compartmentalized, and homogenized to determine perfusion parameters. These parameters serve as input to a poroelastic multi-compartment simulation. The resulting deformation and porosity fields then inform geometry updates and updates to the permeability tensor. To reduce computational costs, we introduce efficient update strategies that avoid costly full re-homogenization at every deformation step while maintaining high accuracy.

The paper is organized as follows: Section 2 reviews the mathematical formulation for generating discrete, non-intersecting vascular trees. Section 3 presents the multi-compartment model for single and multiple vascular trees, including the microcirculation. Sections 4 and 5 discuss parameter estimation based on homogenization principles and the geometric update of model parameters under deformation. Section 6 provides numerical examples to evaluate the accuracy and computational efficiency of our approach. Finally, Section 7 concludes the work and outlines directions for future research.

## Synthetic vasculature: generation of multiple non-intersecting trees

Since in vivo imaging methods are limited in resolution, we generate highly detailed vascular networks synthetically, where it is also possible to incorporate patient-specific vessel data up to the resolution available. We shortly summarize the main aspects of our generation framework based on our previous work (Jessen et al. 2022, 2023), which we recently extended to generate multiple non-intersecting trees inside the same perfusion domain (Jessen et al. 2025).

### Mathematical formulation

A discrete vascular tree is represented as a directed graph $$\mathbb {T}= \left( \mathbb {V}, \mathbb {A}\right) $$ with nodes $$u \in \mathbb {V}$$ and segments $$a \in \mathbb {A}$$. Each segment $$a = uv$$ of $$\mathbb {A}$$ is defined by the geometric locations of its proximal and distal node $$x_u$$ and $$x_v$$, its length $$\ell _a = ||x_u - x_v||$$, volumetric flow $$\hat{Q}_a$$ and radius $$r_a$$. Thus, a $$vessel $$ is simplified to a rigid and straight cylindrical tube. A tree has a single *root* node 0 and (multiple) *leaves* $$v \in \mathbb {L}$$, which are the distal nodes of terminal segments. We assume blood to be incompressible, homogeneous and a Newtonian fluid in the laminar regime. The pressure drop over a segment is described by Poiseuille’s law with

1 $$\begin{aligned} \Delta p_a = \zeta \hat{Q}_a \quad \forall a \in \mathbb {A}, \end{aligned}$$

where the hydrodynamic resistance $\zeta _a$ of each segment *a* is

2 $$\begin{aligned} \zeta _a = \frac{8 \eta }{\pi } \frac{\ell _a}{r_a^4} \quad \forall a \in \mathbb {A}. \end{aligned}$$

We set the dynamic blood viscosity $\eta$ to a (constant) value of 3.6 cP and further assume a homogeneous flow distribution of our (given) root flow $\hat{Q}_\text {perf}$ to all $N_{\text {term}}$ leaves, leading to the terminal flow $\hat{Q}_\text {term} = \hat{Q}_\text {perf}/N_{\text {term}}$. At intermediate branching nodes, the flow can be computed using Kirchhoff’s law with

3 $$\begin{aligned} \hat{Q}_{uv} = \sum _{vw \in \mathbb {A}} \hat{Q}_{vw} \quad \forall v \in \mathbb {V}\setminus \left( {0} \cup \mathbb {L}\right) . \end{aligned}$$

We note that more complex viscosity laws and flow distributions to take into account non-Newtonian behavior of the blood such as the Fåhræus Lindqvist effect can be also incorporated into the framework, see Jessen et al. (2023).

The optimal structure of the final synthetic tree can be based on a combination of different goal functions and constraints, as described in Jessen et al. (2023). Here, we choose to minimize the total power of the tree, which consists of the power to maintain blood inside the vessels $P_\text {vol}$ and the (viscous) power to move blood through vessels $P_\text {vis}$. The cost function for the vascular tree thus becomes

4 $$\begin{aligned} f_\mathbb {T}= P_\text {vol} + P_\text {vis} = \sum _{a \in \mathbb {A}} m_b\pi \ell _a r_a^2 +\frac{8 \eta }{\pi } \frac{\ell _a}{r_a^4}\hat{Q}_a^2, \end{aligned}$$

where $m_b$ is the metabolic demand factor of blood, which we set to ${0.6}\,{\upmu }\hbox {Wmm}^{-3}$ Liu and Kassab (2007). Since we pre-compute all flow values using (3), we can rewrite (4) to

5 $$\begin{aligned} f_\mathbb {T}&= \sum _{a \in \mathbb {A}} w_a(r_a)\ell _a, \end{aligned}$$

where the power weight function $w_a$ is defined at each segment *a* with

6 $$\begin{aligned} w_a(r_a) := w(r_a; m_b, \eta , \hat{Q}_a) := m_b\pi r_a^2 + \frac{8\eta }{\pi r_a^4}\hat{Q}_a^2 \end{aligned}$$

Since our formulation does not include global constraints between nodes, each summand in (5) is decoupled. As shown in Jessen et al. (2025), the globally optimal radius of each segment *a* can then be independently computed with

7 $$\begin{aligned} r_a = \root 6 \of {\frac{16\eta }{m_b\pi ^2}} \root 3 \of {\hat{Q}_a}. \end{aligned}$$

The problem of finding the (globally) optimal tree geometry now only consists of finding the optimal nodal positions *x* and corresponding lengths $\ell$. We seek this optimal geometry using a nonlinear optimization problem (NLP) introduced in Jessen et al. (2022, 2025). Here, we include the nodal positions *x* and the lengths $\ell$ of all segments inside the vector of optimization variables $y = (x, \ell )$. With physical lower bounds $\ell ^-$ and numerical upper bounds $\ell ^+$, the best geometry is found in

8 $$\begin{aligned} Y = \mathbb {R}^{3|\mathbb {V}|} \times [\ell ^-, \ell ^+]^\mathbb {A}, \end{aligned}$$

and our NLP reads:

9 $$\begin{aligned} \min _{y \in Y} \quad&\sum _{a \in \mathbb {A}} w_a\ell _a,\end{aligned}$$

10 $$\begin{aligned} \text {s.t.}\quad&0 = x_u - \bar{x}_u,&u&\in \mathbb {V}_0 \cup \mathbb {L},\end{aligned}$$

11 $$\begin{aligned}&0 = \ell _{uv}^2 - ||x_u - x_v||^2,&uv&\in \mathbb {A}. \end{aligned}$$

where (10) fixes the position $\bar{x}$ of initial nodes $\mathbb {V}_0$ and sampled terminal nodes $\mathbb {L}$ and (11) ensures consistency between nodal positions and segment lengths. Fixing the nodal position $\mathbb {V}_0$ of an initial tree $\mathbb {T}_0$ enables the incorporation of imaging data and can also be extended to fix vessel radii (Jessen et al. 2022; Linninger et al. 2019).

#### Remark

Our current generation method is limited to producing vasculature up to the pre-capillary level. Synthesizing capillary beds would require modifications to the cost function. Such capillary networks have been successfully generated in previous work for the mouse and human brain (Hartung et al. 2021; Linninger et al. 2024).

### Extension to multiple non-intersecting trees

To model the perfusion behavior inside an organ, we need to consider at least two vascular trees; one supplying and one draining tree. Using our optimization problem in (9) for multiple trees independently has a high risk of introducing intersections between vessels. We therefore need to extend the problem by introducing appropriate non-intersection constraints, as highlighted in Jessen et al. (2025). First, we introduce the concept of a vascular *forest* with the index set

12 $$\begin{aligned} \mathbb {F} = \{1,...,N_\mathbb {T}\}, \end{aligned}$$

consisting of $N_\mathbb {T}$ trees $\mathbb {T}_i = (\mathbb {V}_i, \mathbb {A}_i)$. To ensure that these trees do not intersect, we check them pairwise using

13 $$\begin{aligned} \mathbb {P} = \left\{ (i,j): i,j \in \mathbb {F}, i<j \right\} . \end{aligned}$$

For each tree pair $(i,j) \in \mathbb {P}$, we identify the set of quadruples $\mathbb {A}_{ij}$ with

14 $$\begin{aligned} \mathbb {A}_{ij} = \{(a_i, a_j, c_i, c_j) : (a_i, a_j) \in \mathbb {A}_i \times \mathbb {A}_j \text { intersect}\}, \end{aligned}$$

consisting of the set of all pairs $(a_i, a_j)$ of intersecting segments along with their associated nearest points $(c_i, c_j)$. Every quadruple in $\mathbb {A}_{ij}$ with $a_i = u_i w_i$ and $a_j = u_j w_j$ therefore has $c_i \in [u_i, w_i]$ and $c_j \in [u_j, w_j]$ such that

15 $$\begin{aligned} \text {dist}([u_i, w_i], [u_j, w_j]) = \Vert c_i - c_j\Vert < r_{a_i} + r_{a_j}. \end{aligned}$$

We define parameters $\bar{\ell }_{u_i, c_i} := \Vert x_{u_i} - c_i\Vert$ and $\bar{\ell }_{c_i, w_i} := \Vert c_i - x_{w_i}\Vert$, and similarly for $c_j$. Then we add *extension nodes* $v_i$ and $v_j$ (initialized at locations $c_i$ and $c_j$) to split each segment in $\mathbb {A}_{ij}$. Our variable vector $y$ now includes the variables of all individual trees, the locations $x_{v_i}, x_{v_j}$ of the extension nodes, their associated lengths $\ell _{v_i}, \ell _{v_i}$, and the lengths $\ell _{u_i w_i}, \ell _{v_i w_j}$ of split arc pairs. Using the union $A_{\mathbb {P}} := \bigcup _{(i,j) \in \mathbb {P}} A_{ij}$, the extended NLP then becomes:

16 $$\begin{aligned} \min _{y \in Y}&\sum _{i \in \mathbb {F}} \sum _{a_i \in A_i} \ell _{a_i} w_{a_i}(r_{a_i}) \end{aligned}$$

17 $$\begin{aligned} s.t. \quad&0 = x_{u_i} - \bar{x}_{u_i},&i \in \mathbb {F}, \; u_i \in \mathbb {V}_{0,i} \cup \mathbb {L}, \end{aligned}$$

18 $$\begin{aligned}&0 = \ell _{u_i v_i} - \Vert x_{u_i} - x_{v_i}\Vert ^2,&i \in \mathbb {F}, \; u_i v_i \in \mathbb {A}_i,\end{aligned}$$

19 $$\begin{aligned}&0 = \ell _{v_i v_j} - \Vert x_{v_i} - x_{v_j}\Vert ^2,&(a_i, a_j, v_i, v_j) \in \mathbb {A}_{\mathbb {P}},\end{aligned}$$

20 $$\begin{aligned}&\ell _{v_i v_j} \ge r_{a_i} + r_{a_j} + \varepsilon ,&(a_i, a_j, v_i, v_j) \in \mathbb {A}_{\mathbb {P}},\end{aligned}$$

21 $$\begin{aligned}&\ell _{u_i v_i} \ge \bar{\ell }_{u_i c_i}, \; \ell _{u_j v_j} \ge \bar{\ell }_{u_j c_j},&(a_i, a_j, v_i, v_j) \in \mathbb {A}_{\mathbb {P}}, \end{aligned}$$

22 $$\begin{aligned}&\ell _{v_i w_i} \ge \bar{\ell }_{c_i w_i}, \; \ell _{v_j w_j} \ge \bar{\ell }_{c_j w_j},&(a_i, a_j, v_i, v_j) \in \mathbb {A}_{\mathbb {P}}. \end{aligned}$$

The distance between pairs of extension nodes of two trees defined in (19) and (20) ensures that the associated segments do not overlap at these nodes (with a tolerance $\varepsilon$). Lastly (21) and (22) forces segments with extension nodes to develop a kink by preventing the extension nodes from moving closer to their respective proximal or distal node.

### Generation framework

We use the framework described in Jessen et al. (2022, 2025) to generate each tree with a (locally) optimal topology and combine it with the extended NLP to resolve any intersections. First, we sample $N_{\text {term}}$ terminal nodes $\bar{x}$ for each tree inside the (non-convex) perfusion volume (liver) and connect them to the manually set root positions. From these initial (fan) shapes, new topologies are explored by swapping segments. Each *swap* changes the parent nodes between a sampled pair of nodes. The new topology is accepted based on a simulated annealing (SA) approach with probability

23 $$\begin{aligned} P = \exp {\left( \frac{-\Delta f_\mathbb {T}}{T}\right) }. \end{aligned}$$

$\Delta f_\mathbb {T}$ is the change in cost induced by the swap and *T* is the SA temperature. *T* is initialized with a high value, which allows unfavorable (i.e., higher-cost) swaps to be accepted. After each iteration, *T* is gradually decreased ("cooled down") by multiplying it with a factor of 0.9. As a result, the probability of accepting such unfavorable swaps decreases over time. This annealing strategy enables a broader exploration of the topology space than strictly accepting only cost-improving swaps. After a fixed number of swaps, the global geometry is optimized and intersections are resolved as previously explained. Once the trees are generated, we can retrieve all relevant information including the position $x_u$ of each node *u*, and the length $\ell _a$, radius $r_a$, and flow $\hat{Q}_a$ of each segment *a*. Further parameters such as the mean blood velocity $\bar{v}_a = \frac{\hat{Q}_a}{\pi r_a^2}$ can also be computed.

**Fig. 2:**
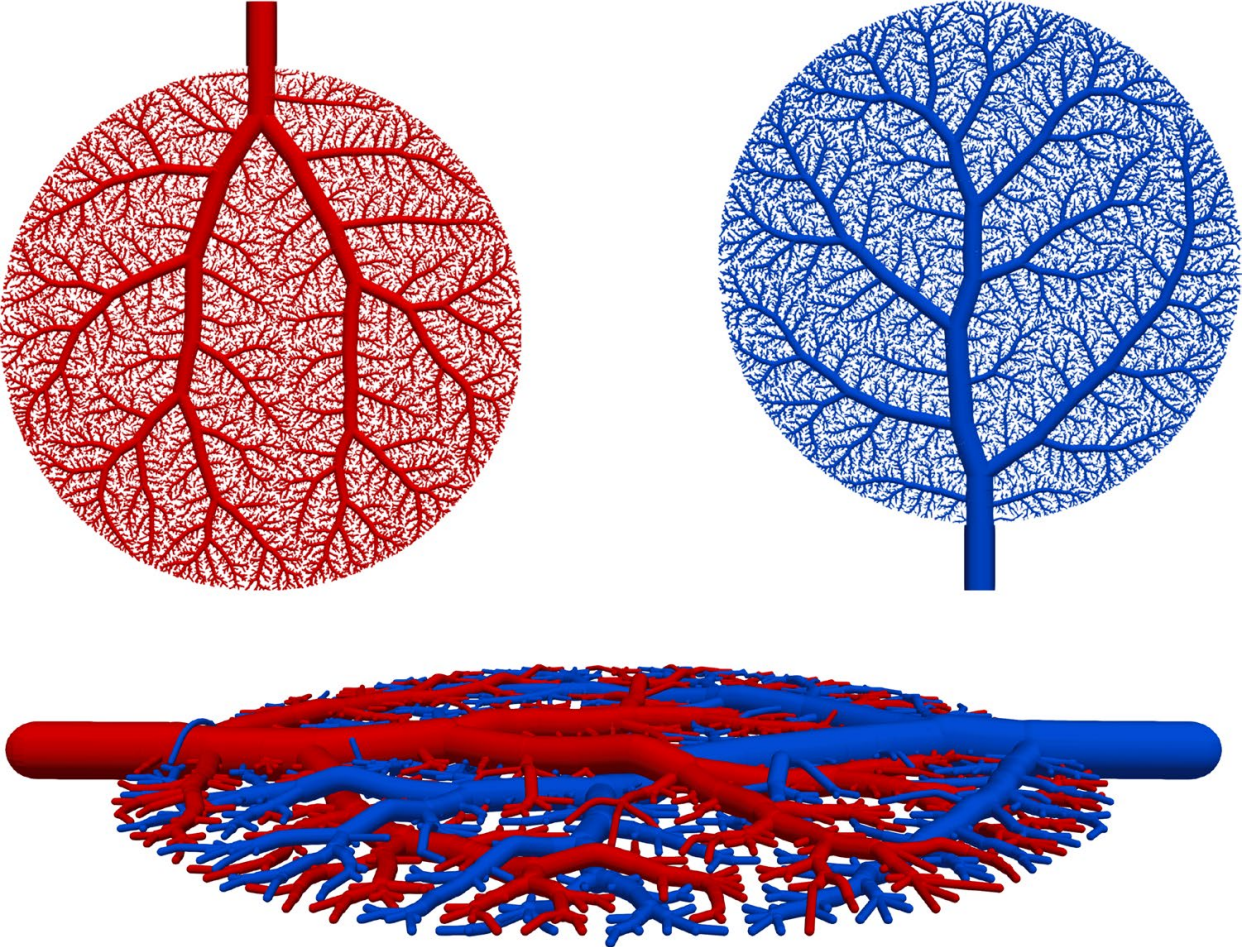
Model problem: supplying (in red) and draining (in blue) vascular trees generated using our generation framework. To avoid intersections between vessels globally, we solve our NLP (16)-(22). Extension nodes are introduced to allow vessels to curve around one another

To illustrate the framework, we consider the problem of supplying and draining a circular perfusion domain $\Omega$ (radius of 10 mm) using two vascular trees. The necessary parameters that characterize both trees are highlighted in Table 1 with their corresponding root positions at opposite ends of the circle. Figure 2 shows the resulting trees. Although all terminal points are located within the planar circular domain, the trees do not intersect, using the third dimension to move their vessel segments around each other.

**Table 1:** Parameters of the discrete vascular structure generated on a circular domain

	$N_\text {seg}$	$N_\text {term}$	$p_\text {root}$ [$\left[ {\frac{{{\mathrm{kg}}}}{{{\mathrm{mm}}{\mkern 1mu} {\mathrm{s}}^{2} }}} \right]$]	$\hat{Q}_\text {perf}$ [$\left[ {\frac{{{\mathrm{mm}}^{3} }}{{\mathrm{s}}}} \right]$]	$m_b$ [$\left[ {\frac{{\mu {\mathrm{W}}}}{{{\mathrm{mm}}^{3} }}} \right]$]	$\eta$ [$\left[ {\frac{{{\mathrm{kg}}}}{{{\mathrm{mm}}{\mkern 1mu} {\mathrm{s}}}}} \right]$]
Supplying tree	43,981	21,991	0.4	80.0	0.6	$3.6 \times 10^{-6}$
Draining tree	43,857	21,929	0.0	80.0	0.6	$3.6 \times 10^{-6}$

## Homogenization of a vascular network via a multi-compartment perfusion model

While direct modeling of blood flow on the vascular tree geometry provided by the discrete model is possible, see, e.g., the technologies reviewed in Formaggia et al. (2010); Caiazzo and Vignon-Clementel (2018), it is computationally expensive. In the following, we present a multi-scale homogenized perfusion model that retains the essential multi-scale characteristics of a hierarchical vascular network while ensuring computational efficiency.

### Compartmentalization of a vascular tree

**Fig. 3:**
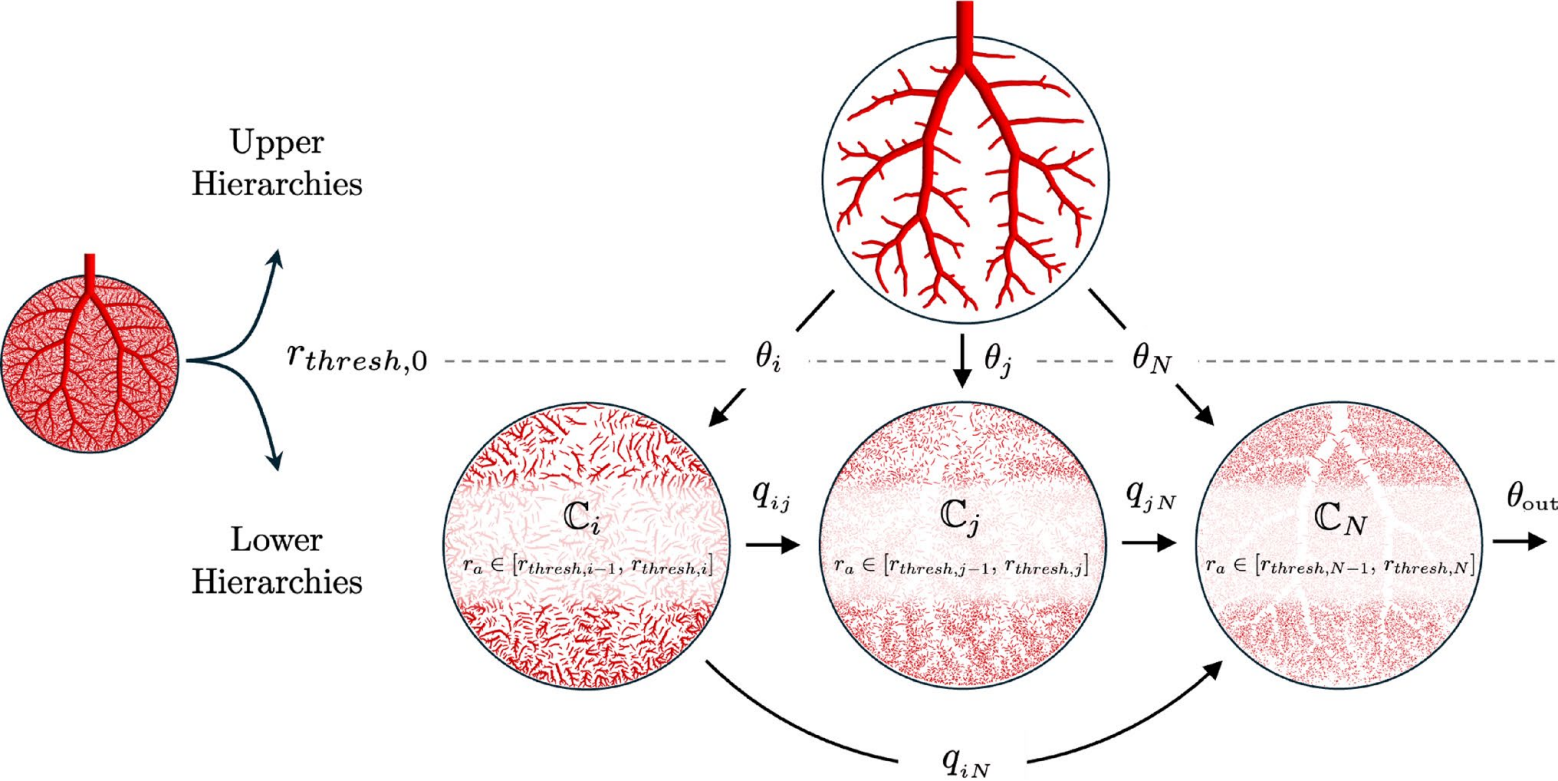
Model structure for single-tree perfusion. The vascular tree is split at the threshold radius $r_{\text {thresh},0}$ into resolved upper hierarchies and homogenized lower hierarchies. The lower hierarchies are divided into *N* compartments, each representing vessels within a specific radius range. The upper hierarchies act as sources, with mass exchange occurring between compartments. Perfusion is ultimately drained through the final compartment $\mathbb {C}_N$. Subscripts *i*, *j*, and *N* denote compartment indices

First-order homogenization relies on the separation of scales, see e.g., Hill (1963); Hornung (2012); Fish (2013). Due to the multi-scale nature of the vascular tree structure, scale separation cannot be guaranteed for all vessels. We assume that vessel segments with radii below or equal to an appropriate threshold of $r_{\textit{thresh,0}}$ are suitable for homogenization. Therefore, we categorize the vessels $a \in \mathbb {A}$ of the vascular tree $\mathbb {T}(\mathbb {V},\mathbb {A})$ in two groups: the *lower hierarchies* suitable for homogenization, and the *upper hierarchies* unsuitable for homogenization and which therefore must remain as a network of resolved vessels. The choice of $r_{thresh,0}$ is problem-specific (see discussion in Sec. 6.1).

#### Remark

In the present work, we employ first-order homogenization, for which scale separation is desirable to ensure the validity of the upscaling process. We note that higher-order homogenization methods exist that can alleviate this requirement by accounting for size effects and higher-order interactions, see e.g., Fish (2013).

The upper hierarchies are represented by the directed graph $\mathbb {C}_0 = (\mathbb {V}_0, \mathbb {A}_0)$ defined by:

24a $$\begin{aligned} \mathbb {A}_{0}&= \{ a\in \mathbb {A}:\, r_a > r_{\textit{thresh},0} \}, \end{aligned}$$

24b $$\begin{aligned} \mathbb {V}_{0}&= \{ u,v\in a=uv:\, a \in \mathbb {A}_{0}\}. \end{aligned}$$

We subdivide the lower hierarchies into *N* compartments. For this, we introduce the ordered set of $N+1$ threshold radii:

25 $$\begin{aligned} \mathbb {D}_{\textit{thresh}} = \left\{ r_{\textit{thresh,0}},\, ...\, , r_{\textit{thresh,N}} \right\} , \quad r_{\textit{thresh},i} < r_{\textit{thresh},i-1}, \end{aligned}$$

and define the compartments $\mathbb {C}_i = (\mathbb {V}_i, \mathbb {A}_i), \, i = 1,..., N$ by:

26a $$\begin{aligned} \mathbb {A}_i&= \{ a\in \mathbb {A}:\, r_{\textit{thresh},i} < r_a \le r_{\textit{thresh},i-1} \},\end{aligned}$$

26b $$\begin{aligned} \mathbb {V}_{i}&= \{ u,v\in a=uv:\, a \in \mathbb {A}_{i}\}, \end{aligned}$$

where we assign all vessels and the respective proximal and distal nodes with radii in a certain range $(r_{\textit{thresh},i}, r_{\textit{thresh},i-1}]$ to compartment *i* (see Fig. 3).

We introduce the subsets of connecting nodes between compartment *i* and *k*:

27 $$\begin{aligned} \mathbb {V}_{i,k}&= \mathbb {V}_{i} \cap \mathbb {V}_{k}, \end{aligned}$$

and define:

28 $$\mathbb {I}_{i,k} = {\left\{ \begin{array}{ll} \{ a=uv \in \mathbb {A}_i \cup \mathbb {A}_k:\, u \in \mathbb {V}_{i,k} \} & \text {if} \; i \ne k,\\ \emptyset & \text {if} \; i = k.\\ \end{array}\right. }$$

### Kinematics and homogenization

**Fig. 4:**
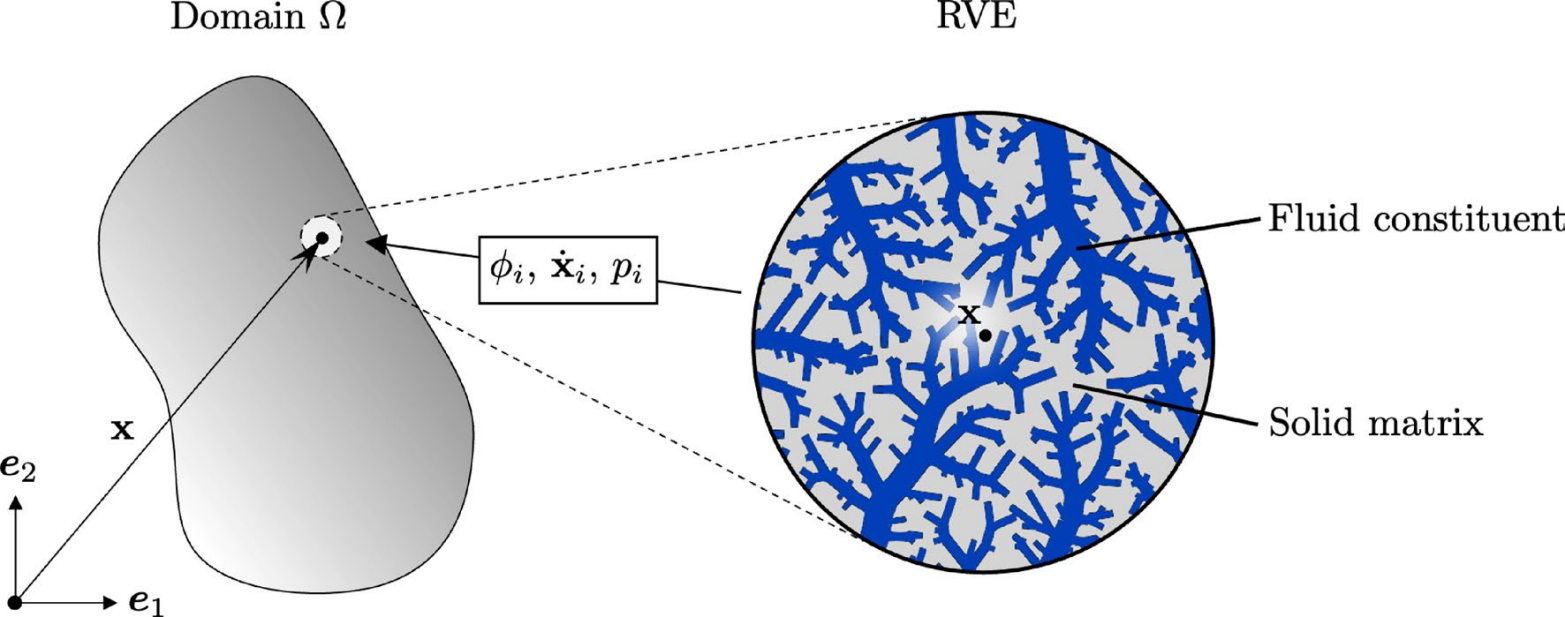
Homogenization concept exemplary for one fluid constituent: macroscopic continuum quantities of constituent *i* and the solid matrix *s*–at a point $\textbf{x}$ in the domain $\Omega$ are defined as spatial averages over the corresponding microscopic fields within the representative volume element (RVE)

To describe blood flow through the lower hierarchical levels of the vascular tree in a homogenized framework, we model the vascularized tissue as a porous medium. For a detailed discussion on the theory of porous media, we refer to Coussy (2004). Here, we focus on fluid flow, assuming a steady state.

We consider an *N*-porosity network, modeling fluid transport based solely on mass balance and Darcy’s law, with the assumption that solid deformation is prescribed. The system consists of $N+1$ constituents: one solid constituent (denoted by index *s*), representing the vessel-surrounding organ tissue, and *N* fluid constituents ($i = 1, \dots , N$), each corresponding to the fluid contained within the vessels of its respective compartment, as defined in the previous section. The fluid in $\mathbb {C}_0$ is treated as an occluded porosity within the solid matrix.

Each constituent is considered a continuum in a homogenized sense. To formalize this, we assume the existence of a representative volume element (RVE), denoted as $V \subset \Omega$, with measure |*V*| and centered at spatial position $\textbf{x}$ (see Fig. 4). Macroscopic continuum quantities are then defined as the spatial averages of the microscopic structure within the RVE. The subvolumes $V_i \subset V$ with measure $|V_i|$ represent the fraction occupied by constituent $i=s,1,\dots ,N$.

#### Remark

In this work, we use the term *Representative Volume Element (RVE)* to denote a subdomain of the vascular structure on which spatial averaging is performed to define macroscopic quantities. Although the concept of an RVE has a precise and rigorous meaning in homogenization theory (see, e.g., Fish (2013)), we do not formally construct or verify statistical representativeness in the strict mathematical sense. Rather, the selected volume is assumed sufficiently large to reflect the characteristic behavior of the microstructure at the associated macroscale point.

We assume that there exists a unique mapping for each of the $N+1$ continuous material bodies $\mathscr {B}_i$ (spatially coexisting in the domain $\Omega$ given by the (invertible) deformation map:

29 $$\begin{aligned} \textbf{x} := \chi _s(\textbf{X}_s,t), \qquad \textbf{x} := \chi _i(\textbf{X}_{i},t), \quad i=1,...,N, \end{aligned}$$

where $\textbf{X}_s$ and $\textbf{X}_{i}$ denote positions in the Lagrangian reference configuration. Provided that scale separation is fulfilled, we can assume that the microstructure deforms homogeneously in the RVE with the macroscopic deformation gradient of the solid;

30 $$\begin{aligned} \textbf{F}_s = \frac{\partial \textbf{x}}{\partial \textbf{X}_s}, \quad J_s = \det (\textbf{F}_s). \end{aligned}$$

A direct consequence of (30) is the transformation rule of the RVE volume:

31 $$\begin{aligned} |V| = J_s |V_0|. \end{aligned}$$

Additionally, we introduce the microscopic velocities:

32 $$\begin{aligned} \textbf{v}_i(\textbf{x},t)=\partial _t\chi _{i}(\textbf{X}_{i},t) |_{\textbf{X}_{i}}, \end{aligned}$$

where we emphasize that $\textbf{X}_i$ is held fixed. Next, we introduce the porosity of constituent *i*:

33 $$\begin{aligned} \phi _i = \frac{|V_i|}{|V|}, \end{aligned}$$

and assume a fully saturated state, i.e., $\phi _s + \phi = 1$, where $\phi =\sum _{i=1}^N \phi _i$ is the total porosity and $\phi _s$ is the volume fraction of the solid matrix.

To proceed, we introduce compartmental values by averaging over the associated RVE. More specifically, denoting by *f* and $\textbf{f}$ arbitrary scalar and vector fields, we define the compartmental averages relative to the compartmental volume $V_i$ and total volume *V*:

34a $$\begin{aligned} \big \langle f \big \rangle _i^*;= \frac{1}{|V_i|}\int _{V_i} f\,\textrm{d}V_i, \quad \text {(real-volume average)}\end{aligned}$$

34b $$\begin{aligned} \big \langle f \big \rangle _i= \frac{1}{|V|}\int _{V_i} f\,\textrm{d}V_i,\quad \text {(bulk-volume average)}\end{aligned}$$

34c $$\begin{aligned} \big \langle \textbf{f} \big \rangle _i= \frac{1}{|V|}\int _{V_i} \textbf{f}\,\textrm{d}V_i, \end{aligned}$$

which are related through the porosity via:

35 $$\begin{aligned} \big \langle f \big \rangle _i =\phi _i \big \langle f \big \rangle _i^*. \end{aligned}$$

Furthermore, for statistically uncorrelated quantities *f* and *g* we have:

36 $$\begin{aligned} \big \langle f g \big \rangle = \big \langle f\big \rangle ^* \big \langle g \big \rangle . \end{aligned}$$

Next, the macroscopic density of constituent *i* is defined as:

37 $$\begin{aligned} \tilde{\rho }_i = \big \langle \rho \big \rangle _i = \rho _i \phi _i. \end{aligned}$$

The macroscopic velocity $\dot{\textbf{x}}_i$ is obtained as the real-volume average of microscopic velocities $\textbf{v}_i$:

38 $$\begin{aligned} \dot{\textbf{x}}_i = \big \langle \textbf{v} \big \rangle ^*_i. \end{aligned}$$

Finally, the filtration velocity (Darcy velocity) is given by:

39 $$\begin{aligned} \textbf{w}_i = \phi _i (\dot{\textbf{x}}_i - \dot{\textbf{x}}_s), \quad i=1,...,N. \end{aligned}$$

#### Remark

The fundamental principles of the *N*-porosity network, as presented above, closely align with the continuum theory of mixtures proposed by Truesdell (1984). Both theories share two key features: (1) the coexistence of *N* constituent bodies, $\mathscr {B}_{i}$ ($i=1,...,N$), occupying the same spatial region simultaneously, and (2) the definition of physical quantities associated with each constituent.

### Multi-compartment model for one vascular tree

Without loss of generality, we first focus on modeling one hierarchical vascular tree in isolation. The corresponding model structure for single-tree perfusion is illustrated in Fig. 3. The upper hierarchies serve as a source, supplying blood to the lower-hierarchy compartments. Mass exchange occurs between compartments, strictly following the hierarchical direction. The network is ultimately drained in the lowest hierarchy compartment, where blood enters the microcirculation.

#### Mass balance

The mass balance is for each fluid compartment ($i=1.,,,.N$) given by:

40 $$\begin{aligned} \partial _t \tilde{\rho }_i + \nabla \cdot (\tilde{\rho }_i \dot{\textbf{x}}_i) = \rho _i (\theta _i - q_i), \end{aligned}$$

where the right-hand side describes the mass transfer between compartments. Here, $\theta _i$ denotes a source term corresponding to the blood supply from the upper hierarchies, and $q_i = \sum _j q_{ij}$ represents mass exchange between compartments, where $q_{ij}$ is the specific mass transport from compartment *i* to *j*. Inserting the Darcy velocity (39), the mass balance becomes:

41 $$\begin{aligned} \partial _t (\tilde{\rho }_i) + \nabla \cdot (\tilde{\rho }_i \dot{\textbf{x}}_s) + \nabla \cdot (\rho _i \textbf{w}_i) = \rho _i (\theta _i-q_i). \end{aligned}$$

Next, we introduce the quantities:

42 $$\begin{aligned} m_i =&~ \tilde{\rho }_i J_s - \tilde{\rho }_{i,0},\end{aligned}$$

43 $$\begin{aligned} \tilde{\rho }_{i,0} =&~ \rho _{i}^f \phi _{i,0}, \end{aligned}$$

where $\phi _{i,0}$ is the initial porosity and $m_i$ denotes the added mass per volume of the reference configuration of compartment *i*. Substitution of (42) provides:

44 $$\begin{aligned} \frac{1}{J_s} \dot{m}_i + \nabla \cdot (\rho _i \textbf{w}_i) =\rho _i (\theta _i-q_i), \end{aligned}$$

where we have employed the identity:

45 $$\begin{aligned} \dot{m}_i = J_s \left( \partial _t \tilde{\rho }_i + \nabla \cdot (\tilde{\rho }_i \dot{\textbf{x}}_s)\right) . \end{aligned}$$

Assuming steady state ($\dot{m}_i =0$) and incompressible fluids ($\rho _i = \textrm{const}$) this simplifies to:

46 $$\begin{aligned} \nabla \cdot \textbf{w}_i +q_i = \theta _i. \end{aligned}$$

##### Remark

In this work, we focus on steady-state mass balance. Modeling dynamic phenomena such as vessel compliance requires retaining the full time-dependent form of the mass balance (44), as employed for example in Barnafi Wittwer et al. (2022) and Montino Pelagi et al. (2024).

The mass balance of the solid compartment satisfies the conservation law:

47 $$\begin{aligned} \partial _t \tilde{\rho }_s + \nabla \cdot (\tilde{\rho }_s \textbf{v}_s) = 0. \end{aligned}$$

The absence of a mass source permits to pull the conservation law back to the reference configuration:

48 $$\begin{aligned} \tilde{\rho }_s J_s = \tilde{\rho }_{s,0} \quad \Leftrightarrow \quad \phi _s J_s = \phi _{s,0}. \end{aligned}$$

#### Homogenized flow equations for one vascular tree

In the final step, we utilize Darcy’s law to formulate the motion of the fluid in the porous medium. For its derivation, we refer the interested reader to Coussy (2004). For the fluid in compartment *i*, we write

$$\begin{aligned} \textbf{w}_i = -\textbf{K}_i \nabla p_i, \end{aligned}$$

where $\textbf{K}_i$ denotes the (positive definite) permeability tensor associated with compartment *i* and $p_i$ the pressure which, in terms of our model, represents the real-volume average in the RVE. With that the total system of first-order differential equations reads:

49a $$\begin{aligned} \textbf{w}_i + \textbf{K}_i \nabla p_i&= \textbf{0} \; \; \;\; \, \text {in} \; \Omega , \end{aligned}$$

49b $$\begin{aligned} \nabla \cdot \textbf{w}_i + q_{i}&= \theta _i \; \;\; \, \text {in} \; \Omega , \end{aligned}$$

in each compartment $i = 1,...,N$, in an open bounded region $\Omega \subset \mathscr {R}^d$, with space dimension *d* and impermeable outer boundary $\Gamma$. We follow the approaches presented in Michler et al. (2013); Hyde et al. (2013) and define $q_{i}$ as a pressure-dependent intercompartmental volumetric flow rate density between compartment *i* and all other compartments (see Fig. 3) and are given by:

50 $$\begin{aligned} q_{i} = \sum _{k=1}^N q_{i,k} = \sum _{k=1}^N \beta _{i,k}(p_i-p_k), \end{aligned}$$

where $\beta _{i,k}\ge 0$ denotes the intercompartmental perfusion coefficient for coupling compartments *i* and *k* and is defined in Sec. 4.3. As such, fluid exchange is absent in pressure equilibrium. Mass balance dictates that the coupling coefficients are symmetric: $\beta _{i,k} = \beta _{k,i}$.

##### Remark

In the related literature, the intercompartmental perfusion coefficient is often referred to as the conductance between compartments.

Summation of (49b) over the compartments $i=1,..., N$ shows that the model (49) locally conserves mass:

51 $$\begin{aligned} \sum _{i=1}^N \nabla \cdot \textbf{w}_i = \sum _{i=1}^N \theta _i \; \;\; \, \text {in} \; \Omega , \end{aligned}$$

where we have invoked the identity:

52 $$\begin{aligned} \sum _{i=1}^N q_i = \sum _{i,k=1}^N \beta _{i,k}(p_i-p_k) = 0. \end{aligned}$$

Substituting (49a) into (49b) converts the first-order system into an equivalent system of one second-order differential equation per compartment:

53 $$\begin{aligned} -\nabla \cdot (\textbf{K}_i \nabla p_i) + q_{i}&= \theta _i \; \;\; \, \text {in} \; \Omega . \end{aligned}$$

Here, the pressure $p_i$ is the sole unknown variable, and the compartment velocities $\textbf{w}_i, i=1,..., N$ follow from Darcy’s law in 49a. We supplement the model with a Neumann boundary condition to account for the impermeability:

54 $$\begin{aligned} -(\textbf{K}_i \nabla p_i) \cdot \textbf{n} = 0 \; \;\; \, \text {on} \; \Gamma _N = \Gamma . \end{aligned}$$

Using the identity $\nabla \cdot \textbf{w}\,d\Omega = \nabla _0\cdot (J_s\textbf{F}_s^{-1}\textbf{w})\,d\Omega _0$ and the pull-back operation $\nabla = \textbf{F}_s^{-T}\nabla _0$, we transform (53) to the Lagrangian formulation:

55 $$\begin{aligned} -\nabla _0 \cdot (\textbf{K}_{i,0} \nabla _0 p_i) + q_{i,0}&= \theta _{i,0} \quad \; \;\; \, \text {in} \; \Omega _0, \end{aligned}$$

with $\nabla _0$ denoting the material gradient and $\textbf{K}_{i,0}, \, \theta _{i,0}=J_s\theta _{i}, \text { and } q_{i,0} = J_s q_i$ denoting the permeability tensor, the source term and the intercompartmental flow rate density in the reference configuration, respectively, where

56 $$\begin{aligned} q_{i,0} = \sum _{k=1}^{N} \beta _{i,k,0}(p_i-p_k), \end{aligned}$$

with $\beta _{i,k,0} = J_s\beta _{i,k}$. The pull-back and push-forward operations for the permeability tensor are, respectively, defined by

57a $$\begin{aligned}&\textbf{K}_{i,0} = J_s \textbf{F}_s^{-1}\textbf{K}_{i}\textbf{F}_s^{-T}, \end{aligned}$$

57b $$\begin{aligned}&\textbf{K}_{i} = J_s^{-1} \textbf{F}_s\textbf{K}_{i,0}\textbf{F}_s^{T}. \end{aligned}$$

The Lagrangian formulation of the Neumann boundary condition (54) reads:

58 $$\begin{aligned} -(\textbf{K}_{i,0} \nabla _0 p_{i}) \cdot \textbf{N} = 0 \; \;\; \, \text {on} \; \Gamma _{N,0} = \Gamma _0, \end{aligned}$$

where $\nabla _0=\textbf{F}_s^T\nabla$ denotes the material gradient and $\textbf{N}$ can be obtained by Nanson’s formula. The model (53) is equipped with the following global properties:

59a $$\begin{aligned} \sum _{i=1}^N \int _\Omega \theta _i~\textrm{d}\Omega =&~ 0,\end{aligned}$$

59b $$\begin{aligned} \sum _{i=1}^N \int _\Omega \theta _i p_i~\textrm{d}\Omega \ge&~ 0. \end{aligned}$$

The property (59a) shows that global mass is conserved, whereas (59b) is a global energy-stability property. The global mass conservation follows from:

60 $$\begin{aligned}&\sum _{i=1}^N \int _\Omega \theta _i~\textrm{d}\Omega = \sum _{i=1}^N \int _\Omega \nabla \cdot \textbf{w}_i~\textrm{d}\Omega = \nonumber \\&\quad -\sum _{i=1}^N \int _\Omega \nabla \cdot (\textbf{K}_i\nabla p_i)~\textrm{d}\Omega = - \sum _{i=1}^N \int _\Gamma (\textbf{K}_i\nabla p_i)\cdot \textbf{n}~ \textrm{d} \Gamma = 0, \end{aligned}$$

where the first identity results from (51). To see (59b) we first note the identity:

61 $$\begin{aligned} \sum _{i=1}^N q_i p_i = \sum _{i,k=1}^N \beta _{i,k} (p_i-p_k)p_i = \frac{1}{2} \sum _{i,k=1}^N \beta _{i,k}(p_i-p_k)^2 \ge 0, \end{aligned}$$

in which we have utilized the symmetry of $\beta _{i,k}$. A straightforward substitution now provides the result:

62 $$\begin{aligned} \sum _{i=1}^N \int _\Omega \theta _i p_i~\textrm{d}\Omega =&~ \sum _{i=1}^N \int _\Omega q_i p_i~\textrm{d}\Omega -\sum _{i=1}^N \int _\Omega \nabla \cdot (\textbf{K}_i\nabla p_i)p_i~\textrm{d}\Omega \nonumber \\ =&~ \sum _{i,k=1}^N \int _\Omega \frac{1}{2} \beta _{i,k}(p_i-p_k)^2 ~\textrm{d}\Omega \nonumber \\&\quad + \sum _{i=1}^N \int _\Omega (\textbf{K}_i \nabla p_i)\cdot \nabla p_i ~\textrm{d}\Omega \nonumber \\&\quad -\sum _{i=1}^N \int _\Gamma p_i(\textbf{K}_i\nabla p_i)\cdot \textbf{n}~ \textrm{d} \Gamma \nonumber \\ \ge&~ 0, \end{aligned}$$

where the boundary term vanishes due to (54).

#### Source and sink terms for mass supply and drainage

Our multi-compartment model is subject to pure zero-flux Neumann boundary conditions (54), which presents two challenges: mass supply must be imposed via source terms, and the pressures $p_i$ can only be determined up to a constant, requiring an additional constraint. To address both issues, we calibrate appropriate source and sink terms in the continuity equation (49b), accounting for mass exchange at the connecting nodes $u \in \mathbb {V}_{0,i}$ and the leaves $v \in \mathbb {L}$. This approach effectively couples the resolved vascular tree structure (upper hierarchies) with the lower-hierarchy compartments, see 3.

To avoid singularities in the homogenized flow results due to point-wise inflow or outlow at $u \in \mathbb {V}_{0,i}$, we set up the following source function

63 $$\begin{aligned} \theta _{i}(\textbf{x}) = \sum _{\begin{array}{c} u\in \mathbb {V}_{0,i} \\ a=uv \end{array}} \frac{\hat{Q}_{a}}{(2\pi \sigma ^{2})^{\tfrac{d}{2}}}\exp {\left( -\frac{1}{2}\frac{\left\Vert \textbf{x}-\textbf{x}_u \right\Vert ^2 }{\sigma ^{2}}\right) }, \end{aligned}$$

where we spatially distribute the net flow $\hat{Q}_{a}$ for each node $u \in \mathbb {V}_{0,i}$ symmetrically in the form of a weighted multivariate Gaussian distribution. We note that $d=\dim (\Omega )$ is the spatial dimension of the problem and $\sigma ^2$ is the variance of the radially symmetric distribution that controls the effective spread of the source distribution. In the following, we will denote $\sigma$ as the source distribution parameter.

##### Remark

In the context of a homogenized model, highly localized source terms are not compatible with the underlying assumptions. A direct transition from discrete inlet points to homogenized flow fields is physically not meaningful and may introduce numerical singularities. Therefore, over-localization of sources—i.e., choosing $\sigma$ too small—should be avoided. Ensuring an adequate representation of source terms in the numerical scheme is essential.

##### Remark

Due to the Gaussian distribution, which has unbounded support, relation (59a) will in general not be exactly satisfied. In this paper, we assume that the source distribution parameter $\sigma$ is chosen sufficiently small such that the corresponding mass error is negligible. As an alternative, one could also scale the Gaussian distribution such that its integration over the finite domain $\Omega$ yields one.

We assume that all terminal nodes $\mathbb {L}$ are exclusively contained in the hierarchically lowest compartment *N*. Specifically, for each terminal node $v \in \mathbb {L}$), we have $v\notin \mathbb {V}_i$ for $i = 1, \dots , N-1$. Therefore, we introduce a pressure-dependent source (or sink) term $\theta _{\text {out}}$ in (63) for the hierarchically lowest compartment *N* of the vascular tree. Similar to the intercompartmental mass exchange, $\theta _{\text {out}}$ is defined as:

64 $$\begin{aligned} \theta _{\text {out}} = -\beta _{N_\alpha ,N_\alpha +1}(p_{N}-p_\text {out}), \end{aligned}$$

where $\beta _{N_\alpha ,N_\alpha +1}$ denotes the intercompartmental perfusion coefficient, accounting for mass exchange at the terminal nodes. The reference pressure $p_\text {out}$ is determined using suitable averaging techniques, e.g., linear interpolation or by taking the average of the terminal pressure values $p_v$, with $\, v\in \mathbb {L}$, within the RVE.

### Multi-compartment model for vascular networks

Full organ modeling necessitates modeling of multiple trees. Organs usually have a supplying and a draining tree. The liver, for instance, has two supplying trees, the hepatic artery and portal vein, and one draining tree, the hepatic vein. Here, we generally extend the previous findings to multiple trees. For this, we define the sets $\mathbb {V}_{i}^{\alpha },\;\mathbb {A}_{i}^{\alpha },\;\mathbb {V}_{i,k}^{\alpha },\;\mathbb {I}_{i,k}^{\alpha }$ for $i,k=0,...,N_\alpha$ according to (24) - (28) for each tree $\mathbb {T}^\alpha ,\;\alpha = 1,...,N^{\mathbb {T}}$ with, respectively, $N_\alpha$ fluid compartments. Each compartment $i = 1,..., N_\alpha$ of the tree $\mathbb {T}^\alpha$ is assigned a (spatially varying) permeability tensor $\textbf{K}^\alpha _i$ and intercompartmental perfusion coefficients $\beta ^{\alpha }_{i,k},\; k = 1,...,N_\alpha +1$ determined by means of the homogenization procedure presented in Section 4.2 and 4.3.

#### Connecting supplying and draining trees with the microcirculation

At the lowest level of the vasculature, blood from a supplying tree enters the microcirculation. Metabolism occurs and blood leaves again via a draining tree. We supplement the model with an additional compartment that accounts for the microcirculation, where all associated quantities are denoted by the index *micro* in the following. The microcirculatory compartment is coupled with the respective vascular tree compartments $N_\alpha$ via the intercompartmental perfusion coefficients $\beta _{N_\alpha ,N_\alpha +1}^{\alpha }$, introduced in 3.3.3. We assume that the microcirculation is supplied or drained only by the hierarchically lowest compartment of the respective vascular tree, therefore:

65 $$\begin{aligned} \beta _{i,N_\alpha +1}^{\alpha } = 0, \quad \forall \,i\ne N_\alpha . \end{aligned}$$

**Fig. 5:**
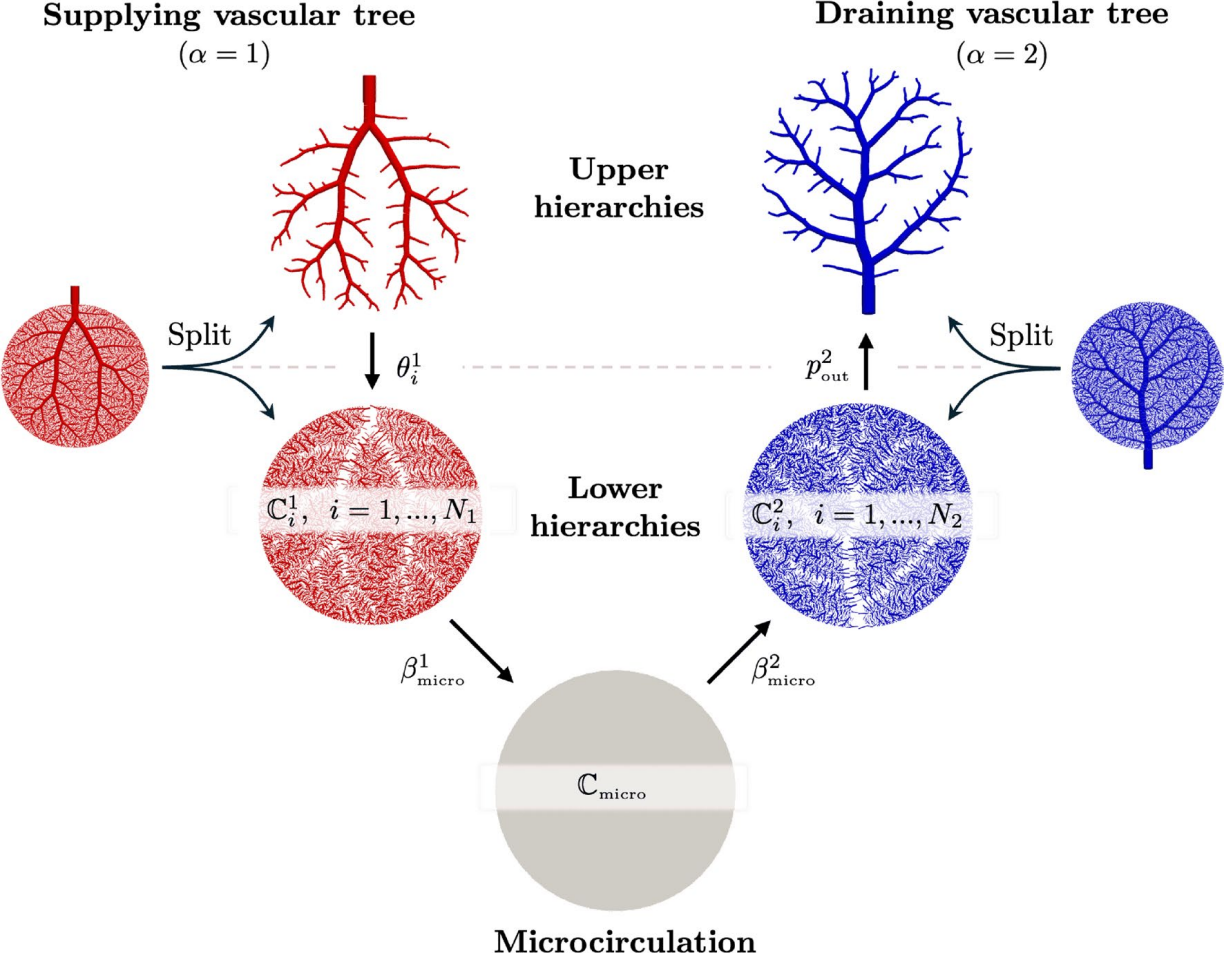
Multi-compartment model structure for a vascular network, including compartments for supplying and draining trees and the microcirculatory compartment

Figure 5 schematically shows the structure of the multi-compartment model for the case of one supplying and one draining tree.

When modeling multiple vascular trees with the multi-compartment model, we must adapt the pressure condition. In contrast to the mass exchange at leaves $v\in \mathbb {L^\alpha }$, where only the lowest compartment $N^\alpha$ contributes, we impose pressure-dependent sink terms $\theta ^\alpha _{i}$ in each compartment *i* of the draining tree $\mathbb {T}^\alpha$ that has a node $u\in \mathbb {V}_{0,i}^{\alpha }$, defined by:

66 $$\begin{aligned} \theta ^\alpha _{i} = -\beta ^\alpha _{\tiny {\text {out}},i}(p^\alpha _{i}-p^\alpha _{\tiny {\text {out}},i}) \; \;\; \, \text {in} \; \Omega ^\alpha _{\tiny {\text {out}},i}, \end{aligned}$$

where $\Omega ^\alpha _{\tiny {\text {out}},i} \subseteq \Omega$,   $\cup _i \Omega ^\alpha _{\tiny {\text {out}},i}\ne \emptyset$ denotes the region of outflow in compartment *i* of draining tree $\mathbb {T}^\alpha$. Again, the prescribed pressures $p^\alpha _{\tiny {\text {out}},i}$ are determined by applying suitable averaging techniques on the nodal pressure values $p_u$ with $u\in \mathbb {V}_{0,i}^{\alpha }$.

We note that one can also model the upper hierarchies with Poiseuille’s law and couple the homogenized compartments by updating $\hat{Q}^\alpha _{u,i}$ and $p^\alpha _{u,i}$ for a supplying and draining tree, respectively. This would result in an iterative procedure to find equilibrium between both models as done in Rohan et al. (2018). However, we assume that the flow characteristics in the upper hierarchies will not change significantly and therefore proceed with a fixed mass supply and outflow pressure.

#### Final system of equations

Finally, our system of equations for the reduced multi-compartment model for $i = 1,...,N_\alpha$ and $\alpha = 1,...,N_\mathbb {T}$ accompanied by the equation for the microcirculation reads

67a $$\begin{aligned}&-\nabla \cdot (\textbf{K}^\alpha _i \nabla p^\alpha _i) + \sum _{k=1}^{N_\alpha +1} \beta ^\alpha _{i,k}(p^\alpha _i-p^\alpha _k) = \theta ^\alpha _i \quad \qquad \qquad \qquad \quad \; \ \text {in} \; \Omega ,\end{aligned}$$

67b $$\begin{aligned}&-\nabla \cdot (\textbf{K}_{\text {micro}} \nabla p_{\text {micro}}) + \sum _{\alpha =1}^{N_\mathbb {T}} \beta _{N_\alpha ,N_\alpha +1}^{\alpha }(p^\alpha _{N_\alpha } -p_{\text {micro}}) = 0 \; \quad \text {in} \; \Omega , \end{aligned}$$

The set of equations in the Lagrangian description then reads:

68a $$\begin{aligned}&-\nabla _0 \cdot (\textbf{K}^{\alpha }_{i,0} \nabla _0 p^{\alpha }_i) + \sum _{k=1}^{N_{\alpha }+1} \beta ^{\alpha }_{i,k,0}(p^{\alpha }_i-p^{\alpha }_k) = \theta ^{\alpha }_{i,0} \quad \qquad \qquad \; \;\; \, \text {in} \; \Omega _0,\end{aligned}$$

68b $$\begin{aligned}&-\nabla _0 \cdot (\textbf{K}_{\text {micro},0} \nabla p_{\text {micro}}) + \sum _{\alpha =1}^{N_\mathbb {T}} \beta _{N_\alpha ,N_\alpha +1,0}^{\alpha } (p^\alpha _{N_\alpha } -p_{\text {micro}}) = 0\; \;\;\text {in} \; \Omega _0. \end{aligned}$$

### Weak formulation and discretization

We utilize the finite element method (Hughes 2012) for the discretization of the reduced multi-compartment Darcy model in (67a,67b). Multiplication with discrete test function $v^\alpha _{h,i}$, integrating over the domain $\Omega$, and subsequently applying integration by parts leads to the weak statement: Find $p^\alpha _{h,i} \in \mathscr {W}^\alpha _{h,i}$ and $p_{h,\text {micro}} \in \mathscr {W}_{h,\text {micro}}$ such that for all $v^\alpha _{h,i} \in \mathscr {W}^\alpha _{h,i}$ and $v_{h,\text {micro}} \in \mathscr {W}_{h,\text {micro}}$:

69a $$\begin{aligned}&\int \limits _{\Omega } \left( \textbf{K}^\alpha _i \nabla p^\alpha _{h,i} \right) \cdot \left( \nabla v^\alpha _{h,i}\right) \, \text {d}\Omega + \int \limits _{\Omega } v^\alpha _{h,i} \sum _{k=1}^{N_{\alpha }+1} \beta ^\alpha _{i,k}(p^\alpha _{h,i}\nonumber \\&\quad -p^\alpha _{h,k}) \, \text {d}\Omega = \int \limits _{\Omega } \theta ^\alpha _i v^\alpha _{h,i} \, \text {d}\Omega , \end{aligned}$$

69b $$\begin{aligned}&\int \limits _{\Omega } \left( \textbf{K}_{\text {micro}} \nabla p_{h,\text {micro}} \right) \cdot \left( \nabla v_{h,\text {micro}}\right) \, \text {d}\Omega \nonumber \\&\quad + \int \limits _{\Omega } v_{h,\text {micro}} \sum _{\alpha =1}^{N_{\mathbb {T}}} \beta ^\alpha _{\text {micro}}(p^\alpha _{h,N_\alpha }-p^\alpha _{h,\text {micro}}) \, \text {d}\Omega = 0, \end{aligned}$$

where subscript *h* denotes the discretized version of the quantity. The discrete function spaces $\mathscr {W}^\alpha _{h,i}$ and $\mathscr {W}_{h,\text {micro}}$ consists of Lagrange basis functions of degree *P*.

Selecting the weighting functions as $v^\alpha _{h,i} = 1,\, v_{h,\text {micro}} = 1$ and $v^\alpha _{h,i}= p^\alpha _{h,i}, v_{h,\text {micro}} = p^\alpha _{h,N_\alpha }$ shows that the discretization inherits the conservation and energy-stability properties (59):

70a $$\begin{aligned} \sum _{\alpha =1}^{N_\mathbb {T}} \sum _{i=1}^{N_\alpha } \int _\Omega \theta ^\alpha _i~\textrm{d}\Omega =&~ 0,\end{aligned}$$

70b $$\begin{aligned} \sum _{\alpha =1}^{N_\mathbb {T}} \sum _{i=1}^N \int _\Omega \theta ^\alpha _i p^\alpha _{h,i}~\textrm{d}\Omega \ge&~ 0. \end{aligned}$$

Note that (70b) implies coercivity of the associated bilinear form:

71 $$\begin{aligned} B(\left\{ p^\alpha _{h,i}\right\} _{i=1,...,N},\left\{ p^\alpha _{h,i}\right\} _{i=1,...,N}) \ge&~ 0, \end{aligned}$$

where the bilinear form is given by:

72 $$\begin{aligned}&B(\left\{ p^\alpha _{h,i}\right\} _{i=1,...,N},\left\{ v^\alpha _{h,i}\right\} _{i=1,...,N}) \nonumber \\&\quad = \int \limits _{\Omega } \left( \textbf{K}^\alpha _i \nabla p^\alpha _{h,i} \right) \cdot \left( \nabla v^\alpha _{h,i}\right) \, \text {d}\Omega \nonumber \\&\quad + \int \limits _{\Omega } v^\alpha _{h,i} \sum _{k=1}^N \beta ^\alpha _{i,k}(p^\alpha _{h,i}-p^\alpha _{h,k}) \, \text {d}\Omega . \end{aligned}$$

#### Remark

For explicitly discretizing the full Darcy formulation (49a) and (49b), one has to satisfy the inf-sup condition (Brezzi and Fortin 2012) due to the saddle point structure of the system. A well-known stable combination is the Taylor–Hood element with a pressure approximation one order lower than the velocity approximation. For a comprehensive comparison of the full and reduced Darcy models in terms of solution time, memory requirements, and accuracy, we refer to Michler et al. (2013).

## Estimation of model parameters

### Microcirculatory permeability

Our framework generates synthetic vasculature up to the pre-capillary level. In the absence of detailed information about the microcirculatory geometry, we adopt common modeling assumptions in which the microcirculatory permeability is treated as homogeneous and isotropic (Michler et al. 2013; Rohan et al. 2018; Berger et al. 2017; Chapelle et al. 2010; MacMinn et al. 2016; Ebrahem et al. 2024; Penta and Ambrosi 2015). This leads to the permeability tensor:

73 $$\begin{aligned} \textbf{K}_{\tiny {\text {micro}}} = \frac{k_{\tiny {\text {micro}}}}{\eta } \textbf{I}, \end{aligned}$$

where $k_{\tiny {\text {micro}}}$ is the intrinsic permeability, $\eta$ the dynamic viscosity, and $\textbf{I}$ the identity tensor. The intrinsic permeability depends on the microstructural geometry and can be estimated using the Kozeny–Carman relation (Kozeny 1927):

74 $$\begin{aligned} k_{\tiny {\text {micro}}} = d^2 \frac{(\phi _{\tiny {\text {micro}}})^3}{1-(\phi _{\tiny {\text {micro}}})^2}, \end{aligned}$$

where *d* is a characteristic length scale. This formulation enables the representation of deformation-induced changes in porosity and, consequently, in permeability. We note that the Kozeny–Carman relation can also incorporate effects of tortuosity through appropriate adjustments, as discussed in Penta and Ambrosi (2015).

#### Remark

The homogenized microcirculation compartment in our model, along with its associated isotropic permeability, represents the hydraulic resistance of the microvascular network. It does not resolve flow within the microcirculation itself and therefore cannot capture distinct flow patterns at the macro- or mesoscale. Instead, the model should be interpreted as describing the averaged redistribution of flow through the microcirculation, mediated by the network of the smallest venules and arterioles.

#### Remark

To realistically model highly complex or anatomically realistic microstructures, experimental measurement or direct computational flow analysis on anatomically resolved microvasculature is preferable. Such microstructures can be obtained from imaging (e.g., ${\upmu }$CT) or synthetically generated (Linninger et al. 2019). Based on these geometries, flow simulations can be performed to extract microcirculatory flow characteristics, as demonstrated in Debbaut et al. (2012) for the human liver and in Linninger et al. (2024); Sweeney et al. (2024) for cerebral tissue. These results may then inform or validate the parameters of the microcirculatory compartment in the the multi-compartment model.

### Permeability tensor of the vascular tree compartments

For the permeability tensors of the vascular tree compartments, we adapt from Huyghe et al. (1989a, 1989b); Huyghe and van Campen (1995a, 1995b) the formulation of the permeability tensor for a network of rigid and straight tubes obeying Poiseuille’s flow. In contrast to these works, we do not introduce a hierarchy parameter as a fourth dimension to account for intercompartmental interaction, since the coupling between the compartments is considered by the introduction of the intercompartmental perfusion coefficient.

We consider the RVE introduced in 3.2 in the current configuration of the domain $\Omega$. The shape of the RVE may change with $\textbf{x}$ according to the deformation gradient of the solid $\textbf{F}_s$.

Our synthetically generated vascular trees consist of straight cylindrical tubes of length $\ell _a$ with constant radius and viscosity. Each vessel segment $a = uv$ can be represented as a parameterized function:

75 $$\begin{aligned} g_a : [0,1] \rightarrow \mathbb {R}^d, \quad s_a \mapsto (1-s_a) \textbf{x}_u + s_a\textbf{x}_v. \end{aligned}$$

We define the index set $\mathbb {A}_i(\textbf{x}) \subset \mathbb {A}_i$, which identifies all vessels in compartment *i* that intersect the RVE:

76 $$\begin{aligned} \mathbb {A}_i(\textbf{x}) = \{ a = uv \in \mathbb {A}_i \mid g_a([0,1]) \cap V \ne \emptyset \}. \end{aligned}$$

If the RVE is chosen as a convex domain in the reference configuration, it remains convex under uniform deformation with the deformation gradient. Consequently, the intersecting vascular segment is characterized by the start and endpoint $\textbf{x}_1$ and $\textbf{x}_2$, which correspond to the parameters $\tau _{a,1}$ and $\tau _{a,2}$ defined as:

77a $$\begin{aligned} s_{a,1}&= \inf \{ s_a\in [0,1] \mid g_a(\tau ) \in V \}, \end{aligned}$$

77b $$\begin{aligned} s_{a,2}&= \sup \{ s_a\in [0,1] \mid g_a(\tau ) \in V \}. \end{aligned}$$

**Fig. 6:**
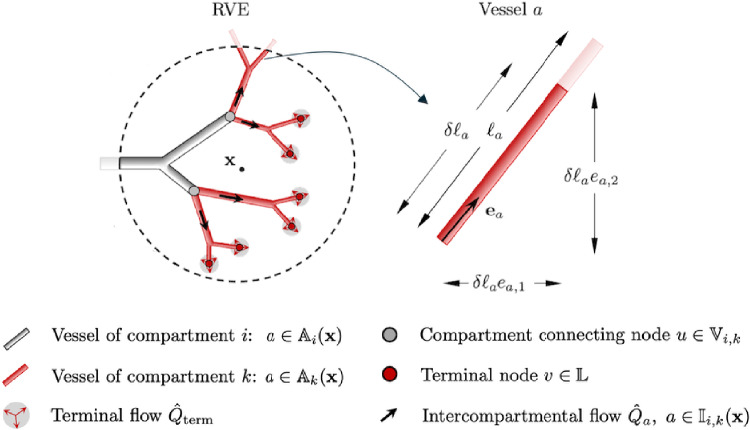
Representative volume element (RVE) intersecting a vascular tree. Left: Vessel segments intersect the RVE at position $\textbf{x}$, forming the set $\mathbb {A}_i(\textbf{x})$. Right: A single vessel segment *a* intersecting the RVE over length $\delta \ell _a$ with orientation $\textbf{e}_a$

These values represent the smallest and largest values of $s_a$ for which the vessel segment intersects the RVE. The intersection span $\delta = s_{a,2} - s_{a,1}$ quantifies the portion of the vessel intersecting the RVE in parameter space. The corresponding intersection points are given by:

78 $$\begin{aligned} \textbf{x}_1 = g_a(s_{a,1}), \quad \textbf{x}_2 = g_a(s_{a,2}), \end{aligned}$$

and give the intersection length of the vessel (see Fig. 6:

79 $$\begin{aligned} \delta \ell _a = \Vert \textbf{x}_2 - \textbf{x}_1 \Vert , \end{aligned}$$

It is related to the initial vessel segment length $\delta L_a$:

80 $$\begin{aligned} \delta \ell _a = \varepsilon _a\, \delta L_a , \end{aligned}$$

where $\varepsilon _a$ denotes the stretch of the vessel element along the vessels center line:

81 $$\begin{aligned} \varepsilon _a = \left\| \textbf{F}_s \textbf{E}_a\right\| = \frac{\textrm{d}s}{\textrm{d}S}, \end{aligned}$$

where $\textbf{E}_a$ is the vessel orientation in the initial state.

**Fig. 7:**
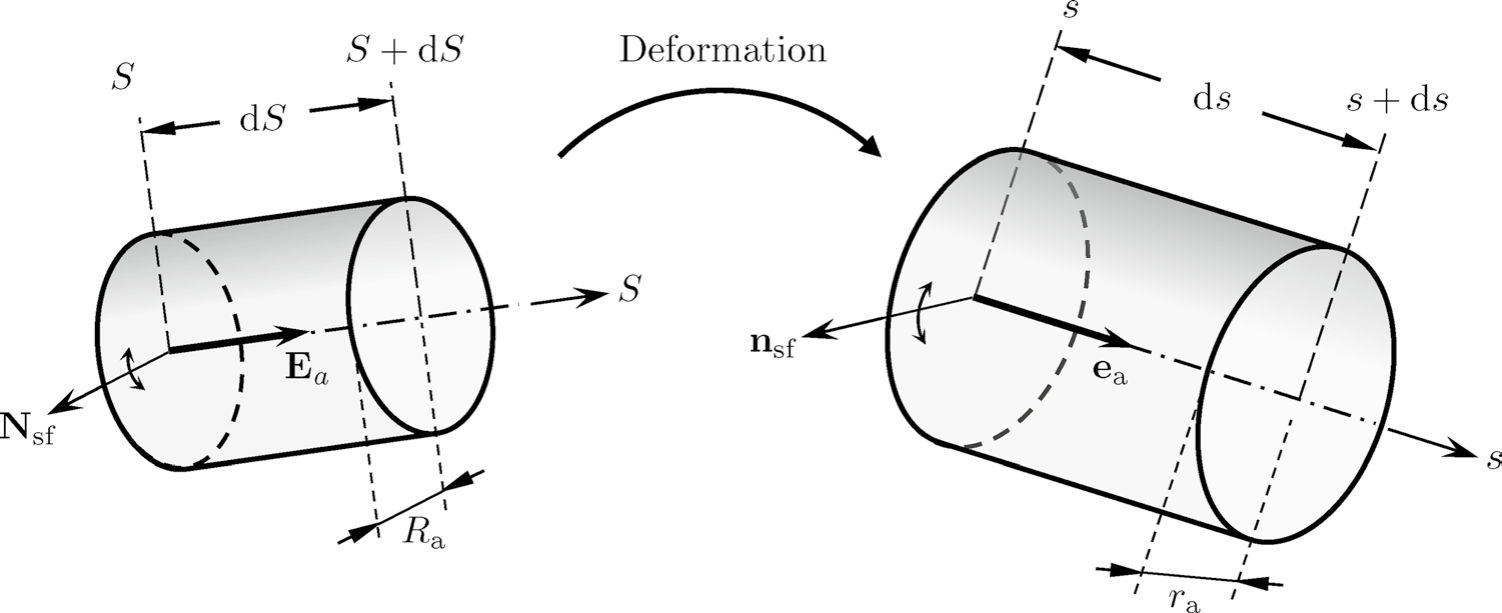
Vessel element in the undeformed state of length $\textrm{d}S$, radius $R_a$ and direction vector $\textbf{E}_a$, and the same Vessel element in the deformed state of length $\textrm{d}s$, radius $r_a$ and direction vector $\textbf{e}_a$

We consider an infinitesimal vascular segment of length $\textrm{d}s$, where the direction unit vector $\textbf{e}_a$ and the vascular segment vector $\textrm{d}\textbf{s}_a$ (see Fig. 7) are related by:

82 $$\begin{aligned} \textrm{d}\textbf{s} = \textrm{d}s \, \textbf{e}_a \quad \Leftrightarrow \quad \textrm{d}s = \Vert \textrm{d}\textbf{s}\Vert = \textrm{d}\textbf{s} \cdot \textbf{e}_a, \end{aligned}$$

where $\Vert \cdot \Vert$ denotes the Euclidean norm. We write Poiseuille’s law for the vessel segment:

83 $$\begin{aligned} \bar{v}_a= - \vartheta _a \frac{\partial p}{\partial s} = - \frac{r_a^2}{8\eta _a}\frac{\partial p}{\partial s}, \end{aligned}$$

with

84 $$\begin{aligned} \vartheta _a = \frac{r_a^2}{8\eta _a}, \end{aligned}$$

where $\bar{v}_a$, $r_a,\, \eta _a, \,\partial p /\partial s$ denote the cross-sectional average velocity of the velocity profile $v_a$, the vessel radius, the dynamic viscosity, and the pressure gradient at position *s* of vessel *a*, respectively.

The pressure gradient with respect to *s* can also be written as a spatial pressure gradient by using the chain rule

85 $$\begin{aligned} \frac{\partial p}{\partial s} = \frac{\partial \textbf{s}}{\partial s} \frac{\partial p}{\partial \textbf{s}} = \textbf{e}_a \cdot \nabla p. \end{aligned}$$

Using (82) and (85) and noting that $\textbf{e}_a$ does not depend on time nor the cross-sectional area, (83) can be rearranged in a straightforward manner to

86 $$\begin{aligned} \bar{\textbf{v}}_a = - \vartheta _a \, \textbf{P}_a \nabla p, \end{aligned}$$

where $\textbf{P}_a = \textbf{e}_a \otimes \textbf{e}_a$ denotes a second-order tensor projecting a vector in the direction of $\textbf{e}_a$.

In (86), $\textbf{v}_a$ already represents the fluid velocity in the vessel segment relative to the surrounding solid. When assuming a quasi-static case, the solid velocity $\dot{\textbf{x}}_s$ is the same for each solid particle. Therefore, by definition in (39) and with (34), (35) and (38), we can find the Darcy velocity by taking the bulk-volume average of the average velocity $\bar{\textbf{v}}_a$ in all vessels $a \in \mathbb {A}_i(\textbf{x})$ in compartment *i*. Since the vascular network is then considered as a one-dimensional Poiseuille flow network embedded in a three-dimensional domain where the velocity is constant in each vessel segment, the Darcy velocity $\textbf{w}$ can be expressed as by means of the adjusted definition of the bulk-volume average:

87 $$\begin{aligned} \textbf{w}_i = \langle \textbf{v}_a \rangle _i = \frac{1}{|V|}\sum _{a\in \mathbb {A}_i(\textbf{x})} \bar{\textbf{v}}_a V_a, \end{aligned}$$

where $V_a= \pi r_a^2 \delta \ell _a$ denotes the volume of the intersecting vessel segment.

To find an expression for the permeability, we apply the spatial averaging theorem that relates the average of the divergence to the divergence of the average (Slattery 1967; Whitaker 1967; Gray 1983; Howes and Whitaker 1985; Whitaker 1999), reading for a vector $\textbf{f}$ in the reference configuration:

88 $$\begin{aligned} \left\langle \nabla _0 \cdot \textbf{f}\right\rangle _0&= \nabla _0 \cdot \left\langle \textbf{f}\right\rangle _0 + \frac{1}{|V_0|} \int _{\Gamma _{\textrm{sf},0}} \textbf{f}\cdot \textbf{N}_{\textrm{sf}}\,\textrm{d}\Gamma _0, \end{aligned}$$

where $\Gamma _{\textrm{sf,0}}$ the interface between the fluid and solid phase within the RVE of volume $|V_0|$ and $\textbf{N}_{\textrm{sf}}$ denoting the unit normal vector on that interface pointing from the fluid into the solid phase (see Fig. 7). In our application, we apply this averaging theorem to vector-valued quantities in direction of the vessel segment. Therefore the boundary term in (88) always vanishes, since:

89 $$\begin{aligned} \textbf{E}_{a,} \cdot \textbf{N}_{\text {sf},a} = 0, \end{aligned}$$

giving:

90 $$\begin{aligned} \left\langle \nabla _0 \cdot \textbf{f}\right\rangle _0&= \nabla _0 \cdot \left\langle \textbf{f}\right\rangle _0. \end{aligned}$$

A condition for the applicability of the spatial averaging theorem is that all RVEs are equal in size, shape, and orientation across all points, while there is no specific constraint on the general shape of the averaging volume. Therefore, we proceed with the Lagrangian form where we define all RVEs to fulfill this criterion. Furthermore, we assume that the vessels remain circular tubes during deformation, giving the following relation for the vessel volume:

91 $$\begin{aligned} \frac{V_{a}}{V_{a,0}}= \frac{r_a^2\,\delta \ell _a}{R_a^2 \, \delta L_a}, \end{aligned}$$

where $R_a$ denotes the initial vessel radius.

The Lagrangian Darcy velocity vector $\textbf{W}=J\textbf{F}^{-1}_s\textbf{w}$ can then be written as:

92 $$\begin{aligned} \textbf{W}_i = \left\langle - \vartheta _{a,0} \textbf{P}_{a,0} \nabla _0 \breve{p}\right\rangle _{i,0}, \end{aligned}$$

where $\langle \,\cdot \,\rangle _0$ denotes the Lagrangian bulk-volume average, $\textbf{V}_a$ denotes the Lagrangian velocity in vessel *a*, $\nabla _0 = \textbf{F}_s^T \nabla$ denotes the material gradient, and $\breve{p}$ denotes the pressure function in the Lagrangian description. $\textbf{P}_{a,0}$ is the projection of a vector on the vessel direction in the reference configuration:

93 $$\begin{aligned} \textbf{P}_{a,0} = {\varepsilon ^2_a} \textbf{F}_s^{-1} \textbf{P}_a \textbf{F}_s^{-T} = \textbf{E}_a \otimes \textbf{E}_a, \end{aligned}$$

and $\vartheta _{a,0}$ is related to $\vartheta _{a}$ by:

94 $$\begin{aligned} \vartheta _{a,0} = \frac{V_a}{\varepsilon _a^2 V_{a,0}} \vartheta _a= \frac{r_a^4 }{8\eta _a\,\varepsilon _a R_a^2 }. \end{aligned}$$

We rewrite (92) by using the product rule and the spatial averaging theorem (90) and obtain:

95 $$\begin{aligned} \textbf{W}_i=\left\langle \nabla _0 \cdot \left[ \vartheta _{a,0}\textbf{P}_{a,0}\right] \, p \right\rangle _{i,0}-\nabla _0 \cdot \left\langle \vartheta _{a,0}\textbf{P}_{a,0} \, p\right\rangle _{i,0}. \end{aligned}$$

Subsequently applying (36) and the product rule to (95) yields:

96 $$\begin{aligned} &  \textbf{W}_i=- \left\langle \vartheta _{a,0} \textbf{P}_{a,0}\right\rangle _{i,0} \nabla _0 \langle p\rangle _{i,0}^* + \left[ \left\langle \nabla _0 \cdot \left[ \vartheta _{a,0}\textbf{P}_{a,0}\right] \right\rangle _{i,0}\right. \nonumber \\ &  \quad \left. - \nabla _0 \cdot \left\langle \vartheta _{a,0}\textbf{P}_{a,0}\right\rangle _{i,0} \right] \langle p\rangle _{i,0}^*, \end{aligned}$$

where the last term vanishes when applying the spatial averaging theorem (88) once more, i.e.

97 $$\begin{aligned} \left\langle \nabla _0 \cdot \left[ \vartheta _{a,0}\textbf{P}_{a,0}\right] \right\rangle _{i,0}=\nabla _0\cdot \left\langle \left[ \vartheta _{a,0}\textbf{P}_{a,0}\right] \right\rangle _{i,0}. \end{aligned}$$

Therefore, (96) reduces to:

98 $$\begin{aligned} \textbf{W}_i= - \left\langle \vartheta _{a,0}\textbf{P}_{a,0}\right\rangle _{i,0} \nabla _0 \langle p\rangle _{i,0}^*, \end{aligned}$$

relating the bulk-volume average of the fluid velocity to the gradient of the real-volume average of the pressure, which we assume to be representative for the pressure at the RVE center point $\textbf{x}$.

Therefore, (98) represents a Darcy-type flow equation in the Lagrangian description:

99 $$\begin{aligned} \textbf{W}_i = - \textbf{K}_{i,0} \nabla _0 \langle p\rangle _{i,0}^*, \end{aligned}$$

with the permeability tensor in the reference configuration:

100 $$\begin{aligned} \textbf{K}_{i,0} = \left\langle \vartheta _{a,0}\textbf{P}_{a,0}\right\rangle _{i,0} = \frac{\pi }{8 |V_0|} \sum _{a\in \mathbb {A}_i(\textbf{x})} \frac{r_a^4 \,\delta L_a}{\eta _a \varepsilon _a } ( \mathbf {E_a} \otimes \mathbf {E_a}) \end{aligned}$$

Applying the push-forward operation defined in (57b) and using (31), (81), and (93), we obtain the permeability tensor in the current configuration:

101 $$\begin{aligned} \textbf{K}_i = \frac{\pi }{8 |V|} \sum _{a \in \mathbb {A}_i(\textbf{x})} \frac{r_a^4 \delta \ell _a}{\eta _a }\left( \textbf{e}_{a} \otimes \textbf{e}_{a}\right) . \end{aligned}$$

#### Remark

The formulations (100) and (101) are equivalent for the initial (undeformed) state and allow the incorporation of the Fåhræus-Lindqvist effect (Fåhræus and Lindqvist 1931) by making $\eta _a = \eta _a(r_a)$ dependend on the vessel radius.

### Intercompartmental perfusion coefficient

Unlike the permeability tensor, which can be determined solely based on geometry, the intercompartmental perfusion coefficients require flow quantities from a reference solution (Hyde et al. 2013). The synthetically generated vascular trees assume Poiseuille flow, providing flow quantities in each vascular segment and the pressure at each node. Using this reference solution, the intercompartmental perfusion coefficient is obtained via

102 $$\begin{aligned} \beta _{i,k}= {\left\{ \begin{array}{ll} \frac{ \left\langle q_{i,k} \right\rangle }{\left| \left\langle p \right\rangle _i^*- \left\langle p \right\rangle _k^*\right| } & \text {if} \;\; \left| \left\langle p \right\rangle _i^*- \left\langle p \right\rangle _k^*\right| \ne 0 ,\\ 0 & \text {else}, \end{array}\right. } \end{aligned}$$

where $\langle q_{i,k}\rangle$ is the bulk-volume average of the intercompartmental flow and $\langle p_i \rangle ^*$ is the real-volume average of the pressure in compartment *i*.

To obtain $\langle q_{i,k}\rangle$, we consider the nodes in the RVE that connect compartments, defined as

103 $$\begin{aligned} \mathbb {I}_{i,k}(\textbf{x})=\{a = uv\in \mathbb {I}_{i,k}: \textbf{x}_u \in V\}, \qquad \mathbb {I}_{i,k}(\textbf{x}) \subset \mathbb {I}_{i,k}. \end{aligned}$$

We then sum the corresponding discrete intercompartmental flow and normalize the result by the volume of the RVE:

104 $$\begin{aligned} \left\langle q_{i,k}\right\rangle = \frac{1}{|V|}\sum _{a\in \mathbb {I}_{i,k}(\textbf{x})}\hat{Q}_a. \end{aligned}$$

We obtain $\langle p \rangle _i^*$ for straight vascular segments with constant diameter via

105 $$\begin{aligned} \left\langle p \right\rangle _i^* = \frac{\sum \limits _{a\in \mathbb {A}_i(\textbf{x})} \left\langle p \right\rangle _a^* \, V_a}{\sum \limits _{a\in \mathbb {A}_i(\textbf{x})} V_a}. \end{aligned}$$

where the average pressure within the vessel segment is defined by:

106 $$\begin{aligned} \langle p \rangle _a^* = \left( 1- \frac{s_{a,1} + s_{a,2}}{2} \right) p_u + \frac{s_{a,1} + s_{a,2}}{2} p_v. \end{aligned}$$

Here, $p_u$ and $p_v$ denote the proximal and distal pressure, respectively, and $s_{a,1}, s_{a,2}$ according to (77).

#### Remark

The case $\left| \left\langle p \right\rangle _i^*- \left\langle p \right\rangle _k^*\right| = 0$ in (102) is not relevant for a hierarchical structured network as the pressure decreases continuously from the root segment to the terminal nodes within the supplying tree, as well as from the terminal nodes back to the root segment within the drainage tree.

## Geometric update of model parameters for deforming vasculature

When the vascular tree deforms, vessel stretching alters both the lengths and radii of individual segments, thereby modifying the hydrodynamic resistance, see (83). In addition, vessels may change orientation. As a result, the permeability in the homogenized multi-compartment model will generally change over time.

For problems that involve large deformations of the vasculature, recalculating the model parameters in each step of a nonlinear solution procedure is computationally expensive. Therefore, we examine strategies to update model parameters, determined once in the initial (undeformed) configuration, using kinematic quantities only.

### General formulation

We assume that a) all vascular segments within the RVE undergo uniform deformation, and b) vascular segments retain their cylindrical shape and show Poiseuille flow after deformation. Vascular segments, defined in the initial configuration by their radius $R_a$, length $\delta L_a$, and orientation $\textbf{E}_a$, are also described in the current configuration by the radius $r_a$, the length $\delta \ell _a$, and orientation $\textbf{e}_a$, see Fig. 7. The current radius of a vessel is generally a (nonlinear) function of the deformation gradient $\textbf{F}_s$ and the initial radius $R_a$:

107 $$\begin{aligned} r_a = r_a (R_a, \textbf{F}_s). \end{aligned}$$

The initial permeability tensor at (pseudo-)time $t=0$ is given by (100) or (101):

108 $$\begin{aligned} \textbf{K}_{i,0}\big |_{t=0} = \textbf{K}_i\big |_{t=0} = \frac{\pi }{8 |V_0|} \sum _{a \in \mathbb {A}_i(\textbf{x})} \frac{R_a^4 \, \delta L_a}{\eta _a\, }\left( \textbf{E}_{a} \otimes \textbf{E}_{a}\right) . \end{aligned}$$

We aim to find an update formulation for the permeability tensors of the form:

109 $$\begin{aligned} \textbf{K}_{i,0} \leftarrow \left( \textbf{K}_i\big |_{t=0},\,\textbf{F}_s\right) , \quad \textbf{K}_i \leftarrow \left( \textbf{K}_i\big |_{t=0},\,\textbf{F}_s\right) . \end{aligned}$$

To this end, we first rewrite (91), assuming that a change in porosity uniformly applies to all vessels inside the RVE, subsequently expand by |*V*| and $|V_0|$ and apply (31), (33):

110 $$\begin{aligned} \frac{r_a^2\,\delta \ell _a}{R_a^2 \, \delta L_a} = \frac{|V_{i}|}{|V_{i,0}|} = \frac{J_s\phi _i}{\phi _{i,0}} \, . \end{aligned}$$

Using (80) and (110), we then find a simple expression for (107):

111 $$\begin{aligned} r_a = \left( \frac{J_s\phi _i}{\varepsilon _a \phi _{i,0}}\right) ^{1/2} R_a \, . \end{aligned}$$

We observe that $r_a$ is linear in $R_a$ and nonlinear in $\textbf{F}_s$. Inserting (111) in (100) yields:

112 $$\begin{aligned} \textbf{K}_{i,0} = \frac{J_s^2 \phi _{i}^2}{\phi _{i,0}^2}\frac{\pi }{8 |V_0|} \sum _{a \in \mathbb {A}_i(\textbf{x})} \frac{R_a^4 \delta L_a}{\eta _a\, \varepsilon ^3_a}\left( \textbf{E}_{a} \otimes \textbf{E}_{a}\right) . \end{aligned}$$

The presence of $\varepsilon ^3_a$ in the denominator in the sum introduces a fundamental challenge for a straightforward update formulation (109), as $\varepsilon _a$ varies for each vessel *a*. Consequently, any computational strategy must explicitly account for these vessel-specific differences, precluding a simple factorization or precomputed update scheme.

### Spectral decomposition and effective vascular segments

We can write the symmetric and positive-definite permeability tensor (101) in the spectral representation as:

113 $$\begin{aligned} \textbf{K}_i = \sum _{n=1}^{\dim \Omega } \lambda _{n,i} \left( \Lambda _{n,i} \otimes \Lambda _{n,i} \right) . \end{aligned}$$

In the context of homogenization, this corresponds to averaging over three *effective vascular segments*, each associated with the orthogonal and normalized eigenvectors $\Lambda _{n,i}$ for $n = 1,..,\dim \Omega$. The eigenvalues can then be defined as functions of the radius $r_{n,i}$ and length $\delta \ell _{n,i}$ of the respective effective vascular segment and the RVE volume |*V*|:

114 $$\begin{aligned} \lambda _{n,i} = \lambda _{n,i} (r_{n,i}, \delta \ell _{n,i}, |V|),\quad \lambda _{n,i} > 0 \,\forall \, n. \end{aligned}$$

The Lagrangian permeability tensor is then given by:

115 $$\begin{aligned} \textbf{K}_{i,0} = \sum _{n=1}^{\dim \Omega } \lambda _{n,i,0} \left( \Lambda _{n,i,0} \otimes \Lambda _{n,0} \right) , \end{aligned}$$

where the initial eigenvectors and eigenvalues satisfy:

116 $$\begin{aligned} \Lambda _{n,i,0} = \varepsilon _{n,i} \textbf{F}_s^{-1} \Lambda _{n,i}, \end{aligned}$$

117 $$\begin{aligned} \lambda _{n,i,0} = J_s \varepsilon _{n,i}^{-2} \lambda _{n,i}. \end{aligned}$$

Here, $\varepsilon _n$ denotes the stretch in the direction of the *n*-th eigenvector. Assuming that the eigenvalues of the effective vascular segments defined in (114) inherit the update properties (31), (80), and (107), we obtain:

118 $$\begin{aligned} \lambda _{n,i} = \frac{\varepsilon _{n,i}}{J_s}\frac{(r_{n,i}(R_{n,i},\textbf{F}_s))^4 }{R_{n,i}^4} \lambda _{n,i} |_{t=0}, \end{aligned}$$

we obtain:

119 $$\begin{aligned} \textbf{K}_i = \frac{1}{J_s} \textbf{F}_s \left( \sum _{n=1}^{\dim \Omega } \frac{(r_{n,i}(R_{n,i},\textbf{F}_s))^4 }{\varepsilon _{n,i}\,R_{n,i}^4} \lambda _{n,i} |_{t=0} \left( \Lambda _{n,i} |_{t=0} \otimes \Lambda _{n,i} |_{t=0} \right) \right) \textbf{F}_s^T. \end{aligned}$$

allowing for the incorporation of physiological models for the radius change by adequately choosing $r_n(R_n,\textbf{F}_s)$. Using the relation (111), this simplifies to:

120 $$\begin{aligned} \textbf{K}_i = \frac{J_s \phi _i^2}{ \,\phi _{i,0}^2} \textbf{F}_s \left( \sum _{n=1}^{\dim \Omega } \frac{\lambda _{n,i} |_{t=0}}{\varepsilon _{n,i}^3} \left( \Lambda _{n,i} |_{t=0} \otimes \Lambda _{n,i} |_{t=0} \right) \right) \textbf{F}_s^T. \end{aligned}$$

The formulation (120) is formally correct under the assumption that all vessels in the representative volume element (RVE) are perfectly aligned with the principal axes of the permeability tensor. In this case, each eigenvector direction directly corresponds to vessels oriented along that axis, and the eigenvalue update reflects the stretch of vessels in that direction. In practice, however, vascular segments within the RVE are distributed over a continuous spectrum of orientations, and alignment with the principal axes is not generally guaranteed. Therefore, the assumption that eigen-vessel stretches accurately represent the deformation of vessels in arbitrary directions is, in general, not fulfilled.

To address this, we subdivide the set of vessels within each RVE into subsets grouped by similar orientations. More precisely, we define a set of orientation intervals $\{\Theta _m\}_{m=1}^{N_\Theta }$ partitioning the orientation domain. Each subset $\mathbb {V}_{\Theta _m}$ then contains all vessel segments whose orientations $\varphi _a$ fall within interval $\Theta _m$:

121 $$\begin{aligned} &  \mathbb {A}_{i,\Theta _m}(\textbf{x}) =\left\{ a\in \mathbb {A}_i(\textbf{x}) \; |\; \varphi _a \in \Theta _m \right\} , \nonumber \\ &  \Theta _m = \bigl [\varphi _m^{\text {min}}, \varphi _m^{\text {max}}\bigr ),\quad \Theta _m\cap \Theta _n = \emptyset \text { for } m\ne n. \end{aligned}$$

For each subset, we compute a dedicated permeability tensor $\textbf{K}_i^{(m)}$ using the spectral approach described above, i.e.,

122 $$\begin{aligned} \textbf{K}_i^{(m)} =\frac{J_s \, \phi _i^2}{\phi _{i,0}^2} \, \textbf{F}_s \left( \sum _{n=1}^{\dim \Omega } \frac{\lambda ^{(m)}_{n,i}|_{t=0}}{\varepsilon ^{(m)\,3}_{n,i}} \left( \Lambda ^{(m)}_{n,i}|_{t=0} \otimes \Lambda ^{(m)}_{n,i}|_{t=0} \right) \right) \textbf{F}_s^{T}. \end{aligned}$$

The total permeability tensor for the RVE is then obtained as the sum over all orientation subsets:

123 $$\begin{aligned} \textbf{K}_i =\sum _{m=1}^{N_\Theta } \textbf{K}_i^{(m)}. \end{aligned}$$

This procedure ensures that the principal stretches used in updating the eigenvalues correspond more closely to the actual vessel deformations in each orientation range. The accuracy of the representation improves as the orientation intervals $\Theta _m$ become narrower, i.e., as $N_\Theta$ increases. However, computational efficiency is maintained by choosing $N_\Theta$ small relative to the total number of vessel segments, resulting in a significant reduction in computational cost. Notably, with this subdivision approach, the number of spectral decompositions and corresponding updates scales as $3 \times N_\Theta$, rather than with the total vessel count. An example of this strategy is demonstrated in Section 6.3.

### Constant hydrodynamic resistance

The update of the vessel radius (107) allows for the incorporation of physiological assumptions into the multi-compartment model. For organs with regenerative capacity, such as the human liver, a simplified update strategy can be introduced. In our model, vessels of the lower hierarchies (i.e., medium- and smaller-sized vessels) are homogenized. In a growing liver, these vessels undergo angiogenesis and remodeling to re-establish connectivity with the existing vascular network, thereby supporting uniform perfusion in the liver (Michalopoulos 2007; Große-Segerath and Lammert 2021). We assume that these biological reorganization effects are implicitly captured by geometric updates during homogenization and can be modeled by adjusting the vessel radius such that the hydrodynamic resistance $\zeta$ of each vessel, given in (2), remains constant over time:

124 $$\begin{aligned} \zeta _a&= \zeta _{a,0}, \end{aligned}$$

125 $$\begin{aligned} \Leftrightarrow \quad r_a^4&= \varepsilon _a R_a^4. \end{aligned}$$

By inserting this relation into (100) and (101), we obtain:

126a $$\begin{aligned} \textbf{K}_0&= \textbf{K} \big |_{t=0}, \end{aligned}$$

126b $$\begin{aligned} \textbf{K}&= J_s^{-1} \textbf{F}_s \, \textbf{K} \big |_{t=0} \textbf{F}_s^T, \end{aligned}$$

demonstrating that, under this assumption, the permeability tensor in the reference configuration remains constant and identical to its initial value. This approach also provides a computationally efficient update scheme, as it enables a simple reformulation that eliminates the need for recalculating model parameters.

Note that using relation (125) for the update of the radii of the effective vascular segments in the spectral decomposition approach (113) yields the exact same update formulation.

#### Remark

We note that this assumption can only be valid over sufficiently large time scales—typically on the order of hours—where vascular remodeling and angiogenesis can occur. Rapid or transient dynamics cannot be captured by this update formulation.

### Intercompartmental perfusion coefficient

The intercompartmental perfusion coefficient is estimated from a reference solution of the discrete Poiseuille network. Since changes in vessel radii across the entire vascular network influence the intercompartmental perfusion coefficient, a simple geometric update is generally not feasible. This challenge can be addressed either by performing a reference computation of the fully resolved Poiseuille network with updated geometry at each time step, or by introducing simplifying assumptions.

We demonstrate this by assuming that each vessel maintains a constant hydrodynamic resistance during deformation. The discrete vessel flow remains unchanged under identical boundary conditions:

127 $$\begin{aligned} \hat{Q}_a = \hat{Q}_{a,0}. \end{aligned}$$

With (31) and (104), we obtain:

128 $$\begin{aligned} \langle q_{i,k} \rangle = J^{-1} \langle q_{i,k}\rangle _0. \end{aligned}$$

Since the nodal pressures in the discrete network remain unchanged, we assume that the average pressure also remains constant, leading to:

129 $$\begin{aligned} \left\langle {p} \right\rangle _i^* = \left\langle {p} \right\rangle ^*_{i,0}. \end{aligned}$$

Thus, the relationship between the initial and current intercompartmental perfusion coefficient is given by:

130 $$\begin{aligned} \beta _{i,k} = J^{-1} \beta _{i,k,0}, \end{aligned}$$

indicating that, in the simplest case, the intercompartmental perfusion coefficient scales inversely with volume change.

## Numerical examples

In the following, we first discuss implications and limitations related to the choice of the RVE size and the vessel radius threshold $r_{thresh,0}$ defined as the maximum vessel radius considered suitable for homogenization. We then consider two benchmark problems, perfusion in a 2D circular disk and in a full-scale liver, to investigate the performance of the multi-compartment modeling approach.

### RVE size and vessel radius threshold

According to homogenization theory, a clear scale separation must exist between the organ scale, the RVE scale, and the fine-scale features to be homogenized. Selecting an appropriate RVE size must balance two competing requirements. On the one hand, the RVE size should be sufficiently large to ensure that scale separation holds and homogenization can be applied. On the other hand, the RVE size should remain small enough to capture relevant macroscale variations.

In theory, the resolution of the underlying synthetic tree is unrestricted from below, allowing the parameter $r_{thresh}$ to be chosen freely. However, in practice, the resolution is constrained by the physiological limit of the smallest vessels in the organ. This physiological limit can be illustrated using the human liver as a representative example. The characteristic length of the human liver, determined, e.g., by the radius of curvature at the notch of the ligamentum teres, the width of the smallest (caudate) lobe or the diameter of the largest vascular segments, is in the range of 1 to 3 cm. The vessel radius threshold $r_{thresh}$ cannot be much smaller than 0.2 mm, as the physiological limit of the smallest arterioles and venules represented in our synthetic tree is around 0.02 mm.

Additionally, larger vessels in the upper hierarchies (in the liver with radii up to 10 mm) act as flow barriers for lower hierarchical levels. These obstructions introduce macroscale variations in the model parameters, necessitating an appropriate choice of RVE radius to capture these effects. Depending on the desired level of detail, the RVE size must be small enough to resolve these macroscale variations while maintaining scale separation. To ensure sufficient scale separation in both directions and adequately capture macroscale variations, the RVE size for the liver cannot be freely chosen, but must be in the limited range of 1 to 5 mm.

We observe that we can achieve a maximum of approximately one order of magnitude of scale separation between the organ scale, the RVE scale, and the fine-scale features to be homogenized. Given these constraints, the RVE size and $r_{thresh}$ can realistically vary by a factor of two or three, which can be expected to have only a limited impact on the model’s outcome.

In this paper, the characteristic length of the macroscale (e.g., the smallest organ-scale diameter), the characteristic length of the RVE (in terms of its radius), and the radii of the largest vessels to be homogenized (specified via the vessel radius threshold $r_{thresh}$) are therefore chosen one order of magnitude apart, although this choice might be considered insufficient according to classical requirements for scale separation in homogenization. We will support our argument by sensitivity studies below.

#### Remark

Given the above arguments, our RVE size is typically larger than the characteristic size of a finite element in our discretization. Hence, for elements located at the domain boundary, the RVE will extend beyond the organ boundary. To address this case, we adapt our averaging approach in the sense of a fictitious domain method (Schillinger and Ruess 2015): we include all vessels within the RVE while adjusting the RVE volume to exclude the portion that lies outside the domain. We note that this modification results in RVEs that differ in shape and size compared to those fully contained within the domain, thereby technically violating the conditions required for the spatial averaging theorem.

**Fig. 8:**
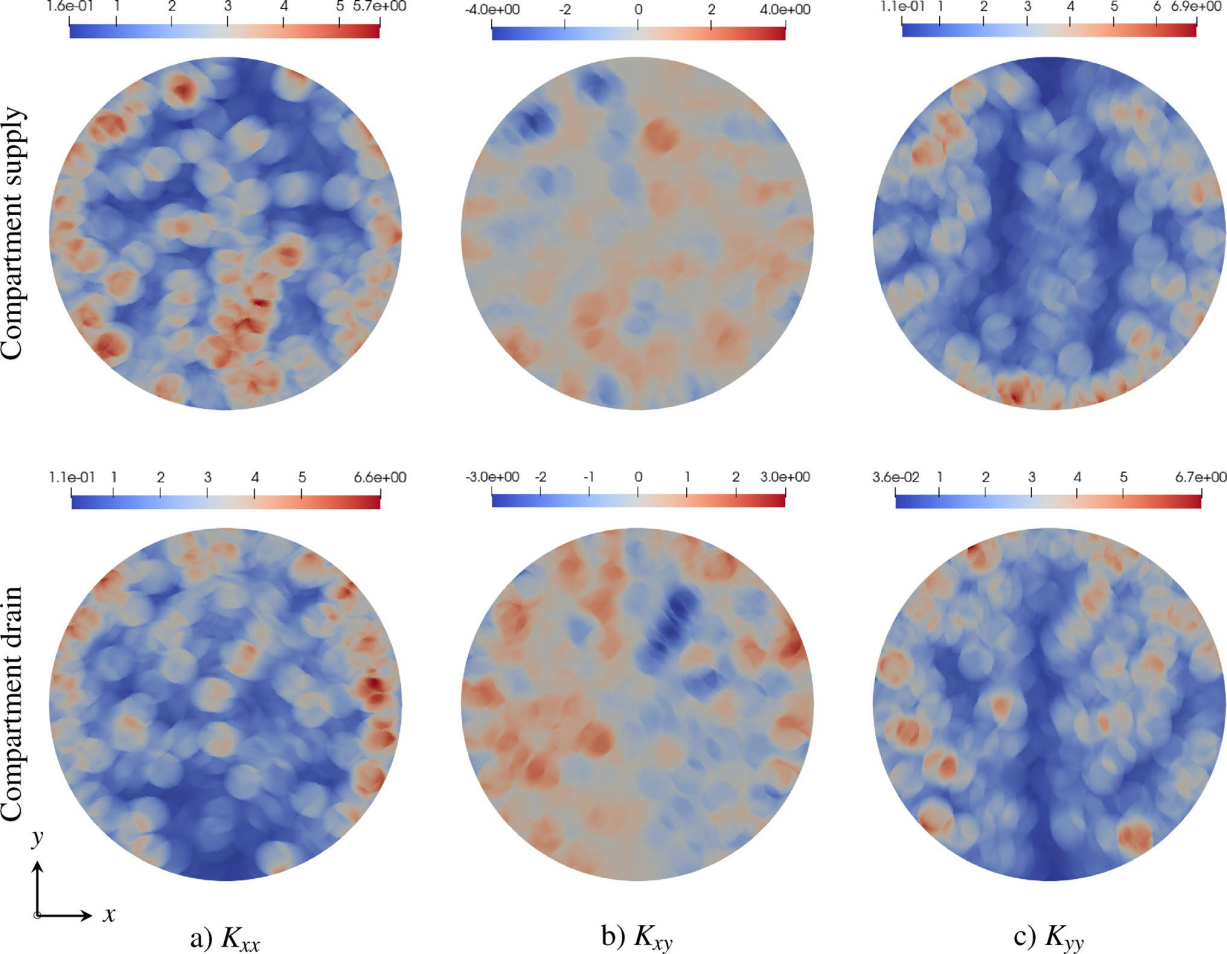
Components of the permeability tensor $\textbf{K}$ in $[\text {mm}^3~\text {s}~\text {kg}^{-1}]$ for the compartments supply (top row) and drain (bottom row), illustrating: a) $K_{xx}$ – permeability in the horizontal direction (flow in *x* induced by a pressure gradient in *x*), b) $K_{xy}$ – coupling term (flow in *x* induced by a pressure gradient in *y*), and c) $K_{yy}$ – permeability in the vertical direction (flow in *y* induced by a pressure gradient in *y*). Note that $\textbf{K}$ is symmetric by construction, i.e., $K_{xy} = K_{yx}$

**Fig. 9:**
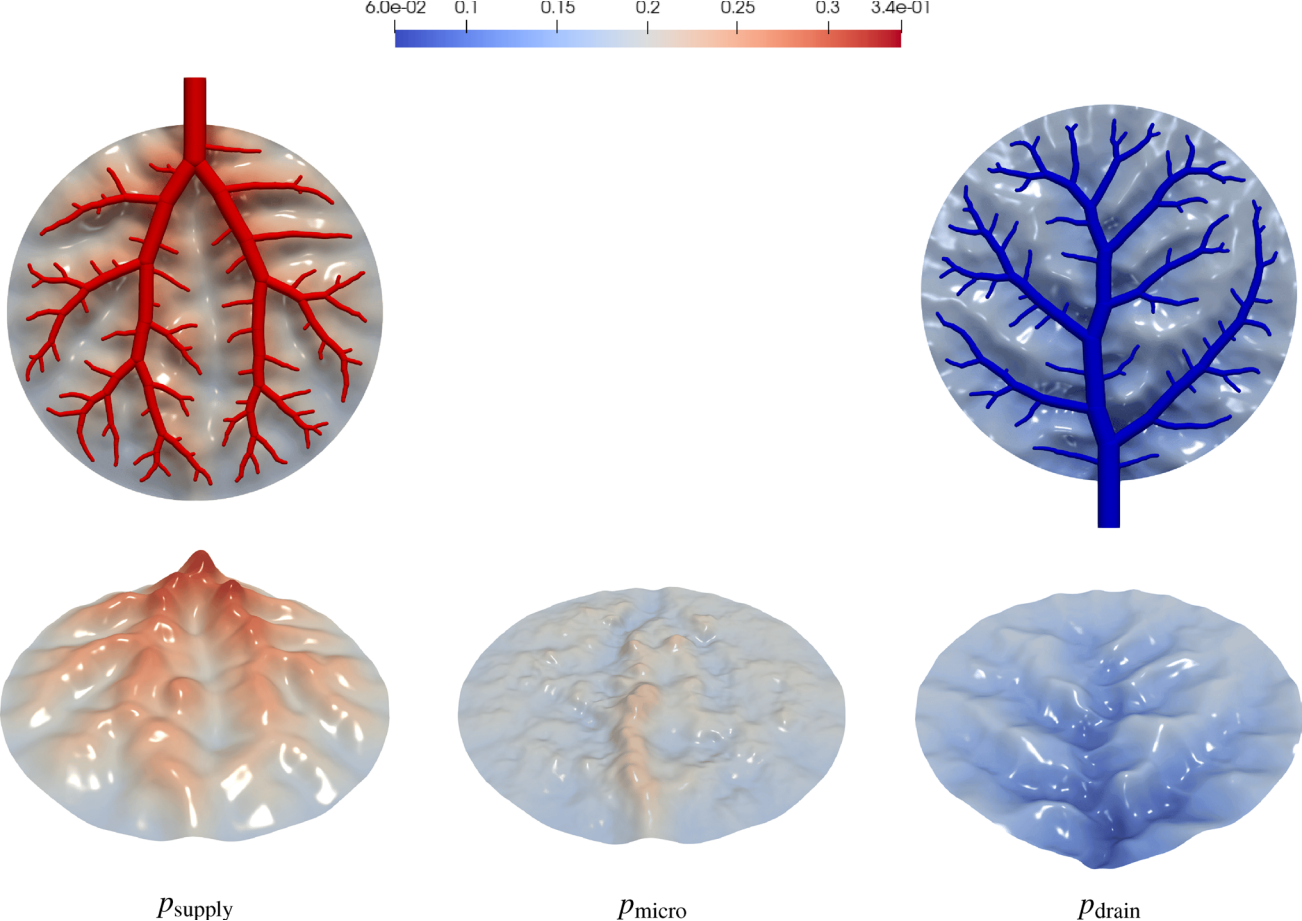
Multi-compartment solution of the pressure fields $p_i$ [$\text {kg} \; \text {mm}^{-1} \; \text {s}^{-2}$]

### Perfusion in a 2D circular disk

We consider the model problem of a 2D circular disk with a radius of $10\,\text {mm}$. Following Section 2, we generate supplying and draining trees, illustrated in Fig. 2. The corresponding parameters are listed in Table 1. Using our compartmentalization strategy ($N_\mathbb {T} = 2$, $N_1 = 1$, $N_2 = 1$), the vasculature is divided into three compartments: $\mathbb {C}^1_1$ (supply), $\mathbb {C}^2_1$ (drain), and $\mathbb {C}_{\text {micro}}$ (microcirculation), as illustrated in Fig. 5. The maximum radius of the vessels to be homogenized is set at $r_{thresh}=0.1\,\text {mm}$, two orders of magnitude smaller than the size of the domain.

For the permeability of the microcirculation, we use the isotropic permeability parameter $k_{\tiny {\text {micro}}} = {2\times 10^{-8}\;\text {mm}^2}$ determined in Debbaut et al. (2012) for human liver tissue. Hence, we obtain an isotropic and homogeneous permeability $K_{\tiny {\text {micro}}} = k_{\tiny {\text {micro}}}/\eta = {1}/{180} \; \text {mm}^3~\text {s}~\text {kg}^{-1}$.

We discretize the domain with 45,955 standard six-noded triangular elements with quadratic basis functions. Each element is assigned a circular RVE centered at its midpoint. Based on the discussion above, the RVE radius is set to 1 mm, ensuring a scale separation of one order of magnitude in both directions and matching the length scale of the largest resolved vessels (maximum diameter: 1.2 mm). The homogenized permeability tensors and intercompartmental perfusion coefficients are assigned to the corresponding elements and assumed constant within each element.

The components of the resulting permeability tensors for the compartments *supply* and *drain* are shown in Fig. 8. The structure of the underlying vascular trees is clearly reflected in the permeability components of the respective compartments. In particular, regions with large vessels of the upper hierarchies are visible–horizontal vessels are prominent in the $K_{xx}$ component, while vertical vessels are captured in the $K_{yy}$ component. These regions exhibit low permeability values due to the absence of lower-hierarchy vessels oriented in the same direction. Additionally, all permeability fields exhibit circular patterns, indicating their dependency on the RVE shape and size. The intercompartmental perfusion coefficient for compartment supply is illustrated in Fig 12 b). We set the standard deviation of the source distribution to $\sigma = 0.6$ mm. The coupled boundary value problem (53)–(54) is solved using the open-source finite element framework FEniCS (Logg et al. 2012).

#### Multi-compartment solution fields

**Fig. 10:**
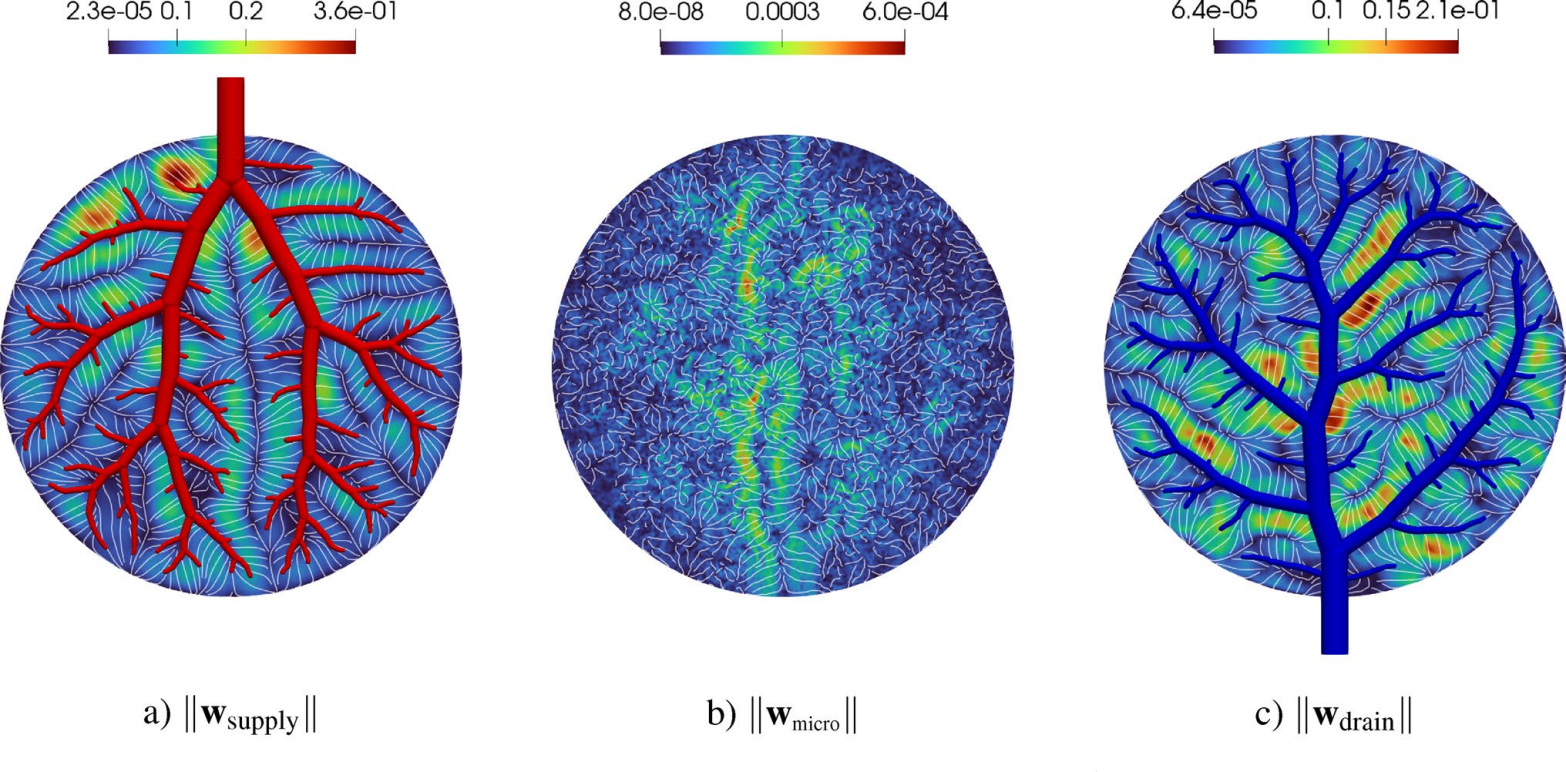
Solution of the homogenized velocity magnitude fields $w_i$ [$\text {mm}\; \text {s}^{-1}$] for **a** the supply compartment, **b** the microcirculation, and **c** the drain compartment. Overlaid streamlines indicate the direction of the homogenized flow fields

**Fig. 11:**
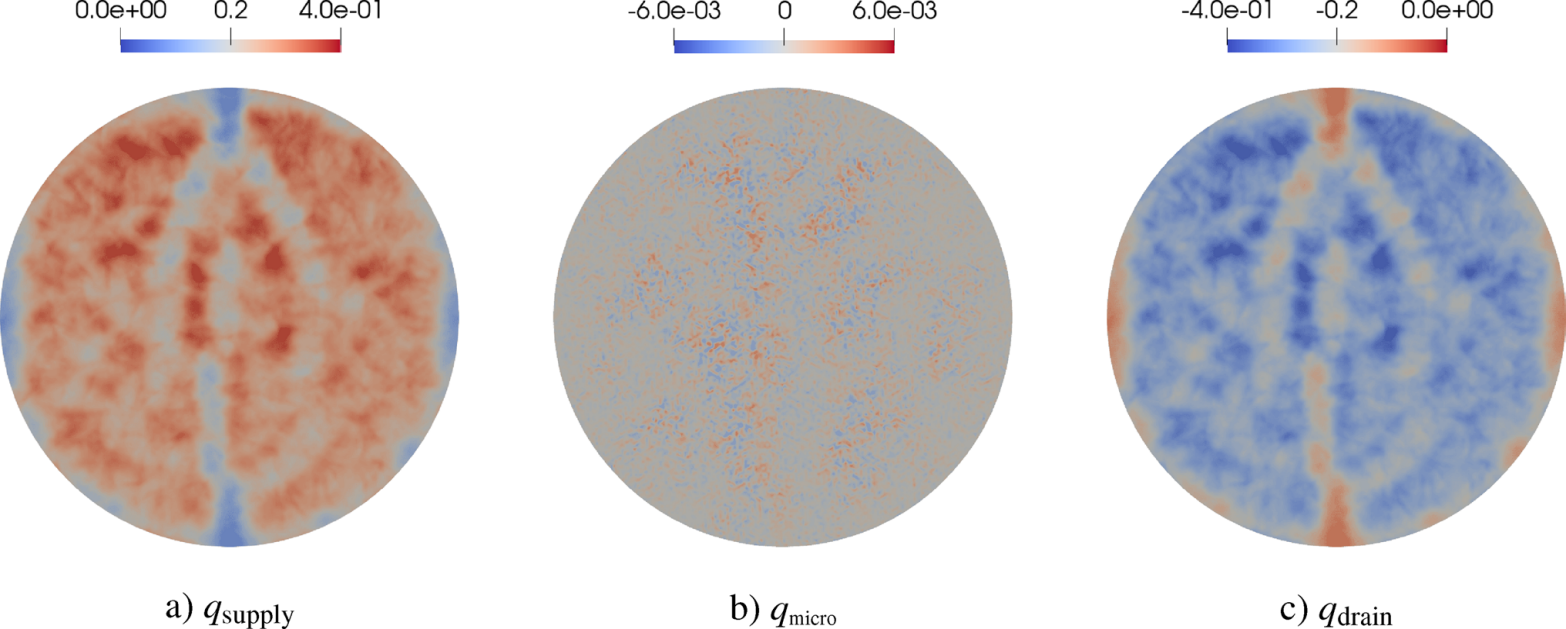
Solution of the homogenized intercompartmental flow rate densities $q_i$ [$\text {s}^{-1}$] representing a) the supply into and c) the drainage from the compartment microcirculation. The difference (b) between supply and drainage shows that the redistribution of fluid within the compartment microcirculation is two orders of magnitude smaller and hence negligibly small

The pressure fields for the three compartments are shown in Fig. 9. The pressure variations in the homogenized compartments of the supplying and draining trees closely follow the structure of the resolved vasculature. In contrast, the microcirculatory compartment displays an almost uniform pressure field. Specifically, pressure in the compartment supply decreases from the root to the terminal branches, while in the compartment drain, it decreases from the terminals toward the root.

Figure 10 plots the velocity magnitudes in all three compartments. Comparing with the resolved vasculature in Fig. 2, we can observe in (a) and (c) that the flow direction aligns with the orientations of the dominant vessels of the lower hierarchies in the respective vascular tree.

In contrast, the microcirculatory compartment does not exhibit an organized flow pattern. The velocity magnitudes are several orders of magnitude smaller than those in the compartments supply and drain. As a result, no significant flow redistribution occurs through the microcirculation compartment and the model does not resolve microcirculatory flow. This finding supports our interpretation that this compartment captures the flow resistance of microcirculation but does not represent substantial flow redistribution at the macroscale.

Figure 11 shows the intercompartmental flow rate densities into and out of the microcirculation. The supply and drainage patterns in a) and c) are nearly uniform and homogeneous. Regions with low intercompartmental flow rate density correspond either to areas where the intercompartmental perfusion coefficient vanishes–typically due to lack of terminals in the presence of larger vessels from the upper hierarchies–or to regions where no significant pressure difference exists between compartments.

The difference between supply and drainage, shown in b), confirms that fluid redistributions within the microcirculatory compartment are negligible and practically do not occur.

#### Sensitivity with respect to RVE size and source distribution parameter

**Fig. 12:**
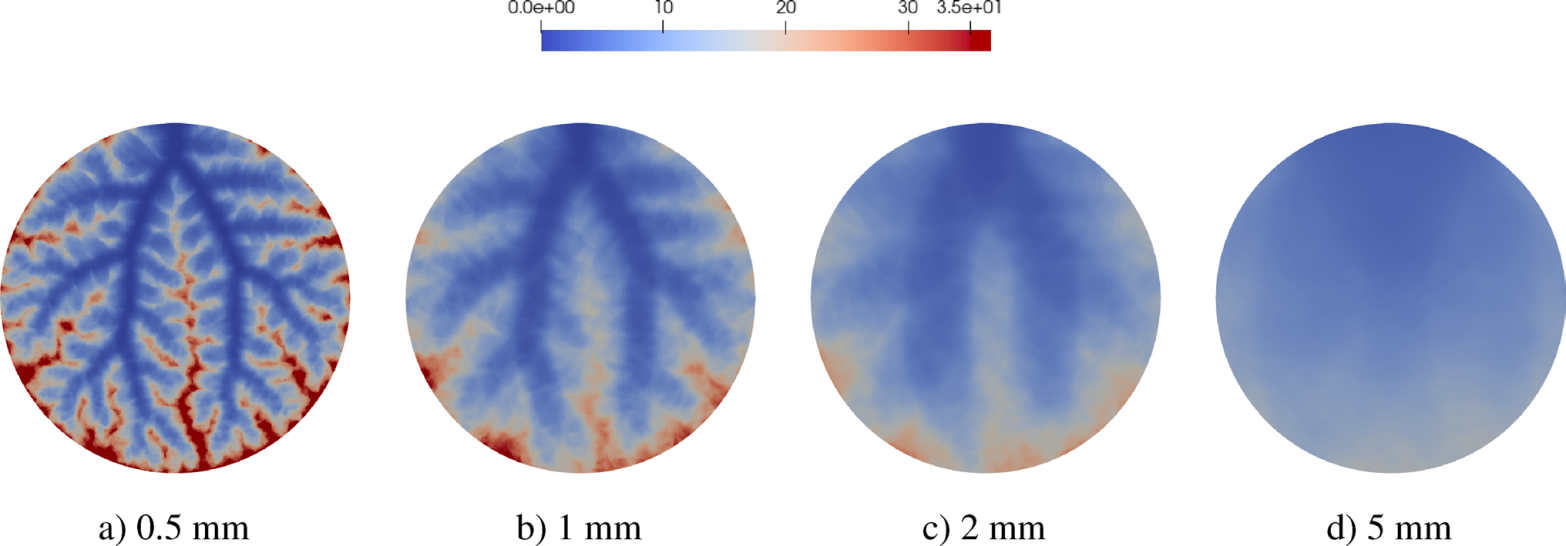
Intercompartmental perfusion coefficient $\beta _{\text {supply}}$ [$\text {mm}~\text {s}~\text {kg}^{-1}$] for different RVE radii. For small RVE radii (0.5 mm and 1 mm), the influence of large vessel geometry and topology is well-resolved. As the RVE radius increases, these structural features are progressively smoothed: partially preserved at 2 mm, but effectively lost at 5 mm

**Fig. 13:**
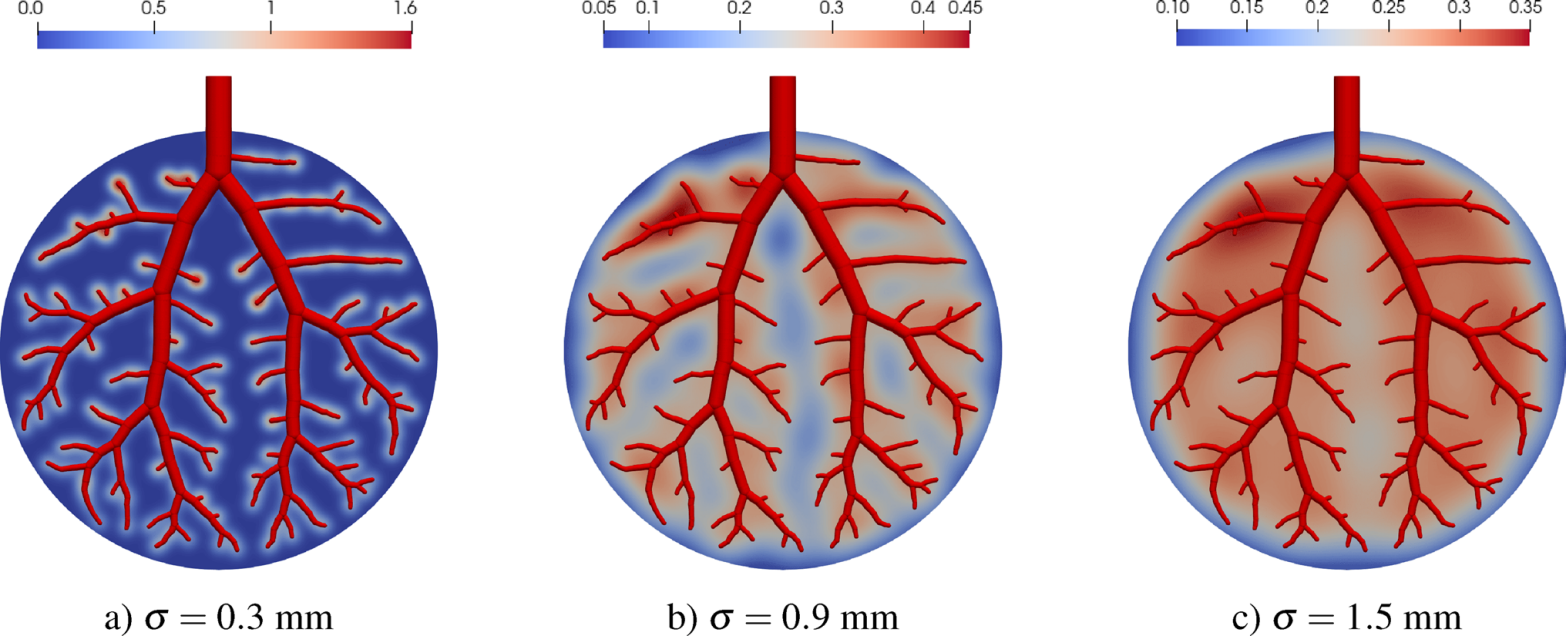
Source distribution $\theta _{\text {supply}}$ [$\text {s}^{-1}$] for different choices of the parameter $\sigma$. As $\sigma$ increases, the distribution becomes more uniform, while smaller values of $\sigma$ result in localized source peaks. This trend is also reflected in the respective color bar ranges. Different graphs indicate variation with respect to the parameter $\sigma$

**Fig. 14:**
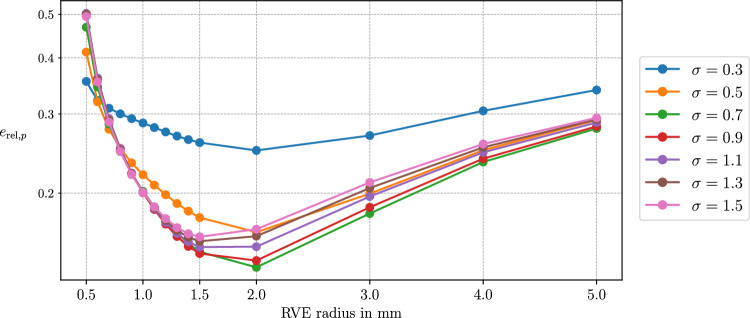
Relative difference in the $L^2$ norm between the homogenized pressure fields obtained for varying RVE radii and the reference pressure field $p_{\text {avg}}$, computed from averaging the discrete pressures of the resolved tree using a reference radius of 1 mm. Different graphs indicate variation with respect to the parameter $\sigma$

**Fig. 15:**
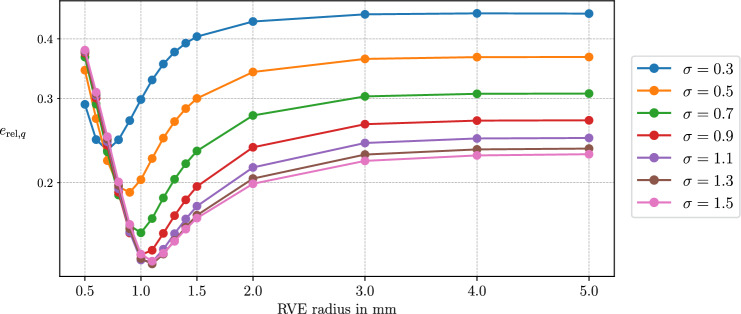
Relative difference in the $L^2$ norm between the homogenized intercompartmental flow rate density and the reference field $q_{\text {avg}}$ (131), computed for varying RVE radii

Figure 12 illustrates the sensitivity of the intercompartmental perfusion coefficient with respect to the RVE radius. For small RVE radii of 0.5 mm and 1 mm, the geometry and topology of the resolved larger vessels are well captured. The resolution of these details is desirable because a) it represents the flow obstruction caused by larger vessels in homogenized compartments and b) it mitigates nonphysical intercompartmental flow between the compartment supply and the microcirculation, and between the microcirculation and the compartment drain at the locations of the larger vessels. As the RVE radius increases, these details become less distinguishable: at 2 mm, the tree structure is smoothed, yet still reflected in the model parameters, whereas at 5 mm, their resolution is entirely lost.

Figure 13 illustrates the sensitivity of the source distribution with respect to the source distribution parameter $\sigma$. For $\sigma = 0.3$ mm, the source term is highly localized at the outlets of the resolved upper tree. As $\sigma$ increases, the source distribution spreads out. For for $\sigma = 0.9$ mm, it still reflects the heterogeneity of the resolved upper vasculature. For $\sigma = 1.5$ mm, the source field approaches a uniform distribution.

For the quantitative analysis, we focus on the compartment supply and perform simulations using the boundary conditions defined in Section 3.3.3. As a reference, we define a pressure field $p_{\text {avg}}$ and compute the intercompartmental perfusion coefficient $\beta _{\text {supply}}$, which is obtained directly from the synthetic vasculature via averaging, using a reference radius of 1 mm as motivated by the preceding observations. We also define a reference field $q_{\text {avg}}$ for the intercompartmental flow rate density as follows:

131 $$\begin{aligned} q_{\text {avg}} = \beta _{\text {supply}}~(p_{\text {avg}}- p_{\text {micro}}) \frac{\hat{Q}_{\text {perf}}}{\int _\Omega \beta _{\text {supply}}~(p_{\text {avg}} - p_{\text {micro})}\,\textrm{d}V}, \end{aligned}$$

representing the spatial distribution of the average intercompartmental flow rate density entering the microcirculation, normalized to $\hat{Q}_{\text {perf}}$. The pressure $p_{\text {micro}}$ corresponds to the outflow pressure $p_{\text {out}}$ in Sec. 3.3.3.

To quantify deviations from the reference fields, we evaluate the relative difference in the $L^2$ norm for the pressure and the intercompartmental flow rate density:

132 $$\begin{aligned} e_{\text {rel,}p} = \frac{\left( \int _{\Omega } (p - p_{\text {avg}})^2\,\textrm{d}V\right) ^{\frac{1}{2}}}{\left( \int _{\Omega } ( p_{\text {avg}})^2\,\textrm{d}V\right) ^{\frac{1}{2}}},\qquad e_{\text {rel,}q} = \frac{\left( \int _{\Omega } (q - q_{\text {avg}})^2\,\textrm{d}V\right) ^{\frac{1}{2}}}{\left( \int _{\Omega } ( q_{\text {avg}})^2\,\textrm{d}V\right) ^{\frac{1}{2}}} \; . \end{aligned}$$

We vary the RVE radius in the homogenization between 0.5 mm and 5 mm and the source distribution parameter $\sigma$ between 0.3 mm and 1.5 mm. For each combination of the two parameters, we compute the pressure *p* and the intercompartmental flow rate density *q* and compare them to the reference fields. The corresponding relative differences in the $L^2$ norm are plotted in Figs. 14 and  15, respectively.

##### Remark

We assume representativity of the homogenized parameters as long as the RVE size remains sufficiently large compared to the finite element size. In this study, all considered RVE sizes are chosen sufficiently large to be representative relative to the mesh resolution.

This sensitivity analysis demonstrates that both the RVE radius and the standard deviation of the source distribution impact the ability of the model to represent the physiological features of interest. For this benchmark case, an RVE radius between 1 mm and 2 mm effectively preserves the influence of larger resolved vessels on permeability and perfusion coefficients, while larger RVE radii lead to a loss of structural information. Similarly, an appropriate choice of $\sigma$, which in this case appears to be around 0.9 mm for this benchmark case, ensures a source distribution that remains consistent with the vascular structure while avoiding over-localization.

### Numerical investigation of permeability updates under deformation

**Fig. 16:**
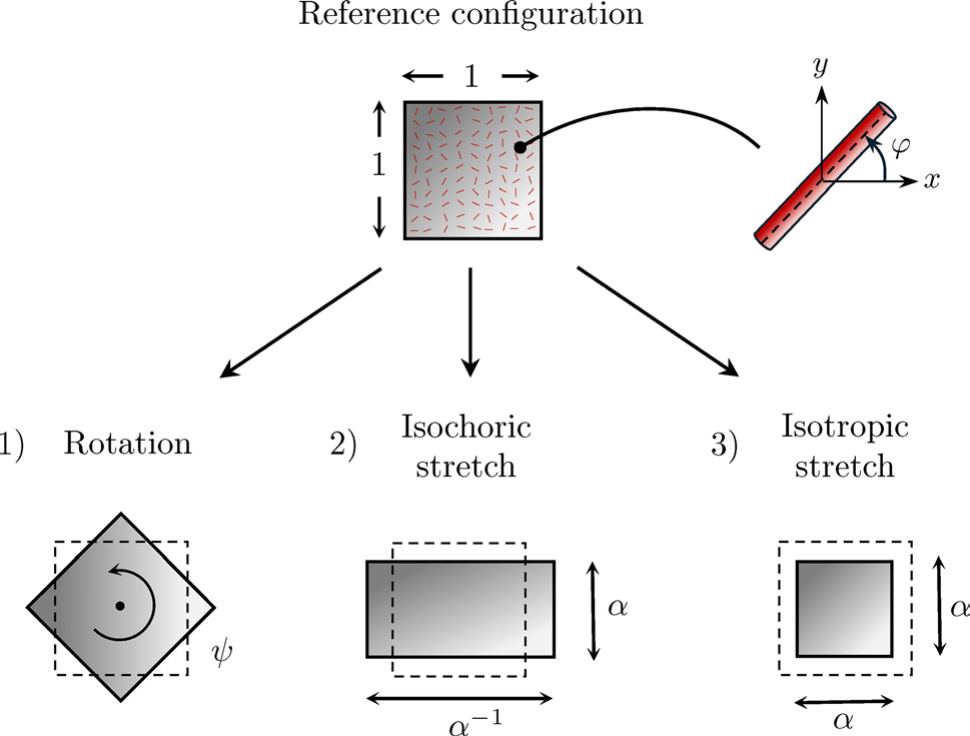
Unit square RVE under three deformation modes. The undeformed RVE is shown together with a vascular segment oriented at angle $\varphi$. The deformation cases are: (1) rotation by $\psi$, (2) isochoric stretch with principal stretches $\alpha$ and $\alpha ^{-1}$, and (3) isotropic stretch with uniform scaling $\alpha$. Dashed outlines indicate the undeformed configuration

This section presents a numerical study of the permeability tensor response under deformation, assessing the accuracy and efficiency of several update strategies against full re-homogenization.

We consider a unit square representative volume element (RVE) containing $N = 100{,}000$ synthetic vascular segments, each with length $\delta L = 0.01$, radius $R_a = 0.01$, and blood viscosity $\eta _a = 10^{-6}$. Two orientation scenarios are studied. In Scenario 1, vessel orientations are randomly distributed within $\varphi \in \left[ \tfrac{\pi }{6}, \tfrac{\pi }{3}\right]$, yielding a strongly anisotropic initial permeability tensor. In Scenario 2, orientations span the entire range $\varphi \in [0,\pi ]$, resulting in an initially isotropic permeability tensor. Throughout all cases, we assume vessels preserve their hydrodynamic resistance under deformation, in order to bring all update formulations to the same baseline and enable a meaningful comparison. Changes in porosity are determined by:

133 $$\begin{aligned} \frac{\phi _i}{\phi _{i,0}} =\frac{V_i}{V_{i,0} \, J_s} =\frac{\displaystyle \sum _{a \in \mathscr {A}_i(\textbf{x})} R_a^2 \, \varepsilon _a^{3/2} \, \delta L_a}{ J_s \displaystyle \sum _{a \in \mathscr {A}_i(\textbf{x})} R_a^2 \, \delta L_a}. \end{aligned}$$

The deformation cases investigated in this numerical experiment are illustrated in Figure 16. Starting from the undeformed unit square RVE, we analyze three fundamental deformation modes: (1) pure rotation of the vascular network by angle $\psi$, (2) isochoric anisotropic stretch with principal stretches $\alpha$ and $\alpha ^{-1}$, and (3) isotropic stretch with uniform scaling $\alpha$. The associated deformation gradients are:

134 $$\begin{aligned} \textbf{F}_{\text {rot}} = \begin{bmatrix} \cos \psi & -\sin \psi \\ \sin \psi & \cos \psi \end{bmatrix}, \quad \textbf{F}_{\text {isochoric}} = \begin{bmatrix} \alpha & 0 \\ 0 & \alpha ^{-1} \end{bmatrix}, \quad \textbf{F}_{\text {isotropic}} = \alpha \, \textbf{I}. \end{aligned}$$

Five update strategies are compared:

1. Re-homogenization assuming uniform porosity change across all vessels (Eq. (112) + push forward).
2. Re-homogenization with vessel-wise update under constant hydrodynamic resistance (Eqs. (101) with (125)).
3. Spectral decomposition without orientation subdivision and uniform porosity update (Eq. (120)).
4. Spectral decomposition with $N_\Theta = 10$ angular subintervals, computing permeability tensors per subset, and subsequently summing the contributions to obtain the total permeability tensor (Eq. (123)).
5. Simple analytical update applying constant hydrodynamic resistance directly to the tensor without re-homogenization (Eq. (126b)).

**Table 2:** Summary of permeability updates for Scenario 1 vessels oriented within $\varphi \in \left[ \tfrac{\pi }{6}, \tfrac{\pi }{3}\right]$.

Deformation	Method	$K_{xx}$	$K_{xy}$	$K_{yy}$	Computation time [s]
Undeformed	Homogenization	1.9982e+00	1.8746e+00	1.9288e+00	$5.01\text {e}{+03}$
Rotation $\psi =\pi /4$	Re-homogenization (uniform)	8.8921e$-$02	3.4672e$-$02	3.8381e+00	$6.03e+00$
	Re-homogenization (vessel)	8.8921e$-$02	3.4672e$-$02	3.8381e+00	$5.91e+00$
	Spectral (no subdivision)	8.8921e$-$02	3.4672e$-$02	3.8381e+00	$3.22e-04$
	Spectral (with subdivision)	8.8921e$-$02	3.4672e$-$02	3.8381e+00	$1.55e-03$
	Simple analytical update	8.8921e$-$02	3.4672e$-$02	3.8381e+00	$3.80e-05$
Isochoric stretch $\alpha =2/3$	Re-homogenization (uniform)	4.3937e+00	2.0045e+00	1.0005e+00	$6.12e+00$
	Re-homogenization (vessel)	4.4959e+00	1.8746e+00	8.5726e$-$01	$5.82e+00$
	Spectral (no subdivision)	4.4634e+00	1.8578e+00	8.5111e$-$01	$6.55e-04$
	Spectral (with subdivision)	4.3945e+00	2.0033e+00	9.9900e$-$01	$1.37e-03$
	Simple analytical update	4.4959e+00	1.8746e+00	8.5726e$-$01	$3.90e-05$
Isotropic stretch $\alpha =2/3$	Re-homogenization (uniform)	1.9982e+00	1.8746e+00	1.9288e+00	$5.82e+00$
	Re-homogenization (vessel)	1.9982e+00	1.8746e+00	1.9288e+00	$5.66e+00$
	Spectral (no subdivision)	1.9982e+00	1.8746e+00	1.9288e+00	$3.79e-04$
	Spectral (with subdivision)	1.9982e+00	1.8746e+00	1.9288e+00	$7.86e-04$
	Simple analytical update	1.9982e+00	1.8746e+00	1.9288e+00	$3.19e-05$

The table reports computed components of the permeability tensor and computational times for each deformation mode and update method

**Table 3:** Summary of permeability updates for Scenario 2 with vessels oriented within $\varphi \in [0,\pi ]$.

Deformation	Method	$K_{xx}$	$K_{xy}$	$K_{yy}$	Computation time [s]
Undeformed	Homogenization	1.9623e+00	−4.5094e$-$03	1.9647e+00	5.16e+00
Rotation $\psi =\pi /4$	Re-homogenization (uniform)	1.9680e+00	−1.2074e$-$03	1.9590e+00	5.93e+00
	Re-homogenization (vessel)	1.9680e+00	−1.2074e$-$03	1.9590e+00	5.85e+00
	Spectral (no subdivision)	1.9680e+00	−1.2074e$-$03	1.9590e+00	2.92e$-$04
	Spectral (with subdivision)	1.9680e+00	−1.2074e$-$03	1.9590e+00	7.44e$-$04
	Simple analytical update	1.9680e+00	−1.2074e$-$03	1.9590e+00	2.79e$-$05
Isochoric stretch $\alpha =2/3$	Re-homogenization (uniform)	3.3594e+00	−3.5818e$-$03	2.2675e+00	6.00e+00
	Re-homogenization (vessel)	4.4151e+00	−4.5094e$-$03	8.7320e$-$01	5.93e+00
	Spectral (no subdivision)	4.1639e+00	−4.9617e$-$01	9.4059e+01	2.21e$-$04
	Spectral (with subdivision)	3.3607e+00	−3.7484e$-$03	2.2661e+00	1.29e$-$03
	Simple analytical update	4.4151e+00	−4.5094e$-$03	8.7320e+01	5.67e$-$05
Isotropic stretch $\alpha =2/3$	Re-homogenization (uniform)	1.9623e+00	−4.5094e$-$03	1.9647e+00	5.96e+00
	Re-homogenization (vessel)	1.9623e+00	−4.5094e$-$03	1.9647e+00	5.72e+00
	Spectral (no subdivision)	1.9623e+00	−4.5094e$-$03	1.9647e+00	3.62e$-$04
	Spectral (with subdivision)	1.9623e+00	−4.5094e$-$03	1.9647e+00	8.01e$-$04
	Simple analytical update	1.9623e+00	−4.5094e$-$03	1.9647e+00	2.50e$-$05

The table reports computed components of the permeability tensor and computational times for each deformation mode and update method

Table 2 shows that in the undeformed state, the permeability tensor reflects strong anisotropy, as indicated by the nonzero off-diagonal component $K_{xy}$. Under rotation, vessels reorient toward the $y$-direction, resulting in increased $K_{yy}$ and reduced $K_{xx}$ and $K_{xy}$. The isochoric stretch leads to an increase in $K_{xx}$, associated with the stretched direction, and a decrease in $K_{yy}$ and $K_{xy}$, as vessels align along the stretch axis and enlarge their radius under constant hydrodynamic resistance. Isotropic stretch causes no change in permeability since all vessels are uniformly deformed without rotation.

All update schemes accurately capture rotation and isotropic stretch. For isochoric stretch, small differences appear between methods, stemming from whether porosity changes are uniformly distributed across vessels or tracked vessel-wise. Overall, deviations remain below $2\%$ for $K_{xx}$, although differences in $K_{yy}$ can reach up to $15\%$. Notably, the spectral decomposition with orientation subdivision achieves deviations below $0.2\%$ compared to uniform re-homogenization. The simple analytical update matches exactly the vessel-wise re-homogenization results, confirming its efficiency and precision under constant hydrodynamic resistance. All methods are suitable for updating anisotropic permeability, but computationally efficient schemes like spectral decomposition (with or without subdivision) are preferable.

For Scenario 2 (Table 3), the initial permeability tensor is nearly isotropic, with $K_{xx} \approx K_{yy}$ and negligible $K_{xy}$. Under rotation, no significant changes occur in the permeability components, reflecting the isotropic nature of the vessel distribution. Likewise, isotropic stretch yields no change across all methods. The permeability remains unchanged across all methods, confirming that uniform scaling does not affect the permeability tensor when vessels preserve their hydrodynamic resistance and orientation distribution.

Significant differences arise under isochoric anisotropic stretch. Re-homogenization with uniform porosity change differs considerably from re-homogenization with vessel-wise geometry update, as the latter concentrates volume changes in vessels aligned with the deformation direction. This results in notable discrepancies between these approaches. The spectral decomposition without orientation subdivision fails to capture the anisotropy induced by deformation. In contrast, spectral decomposition with orientation subdivision achieves excellent agreement with the re-homogenization results, achieving deviations below $0.07\%$ in $K_{xx}$ and $K_{yy}$, and around $4\%$ in $K_{xy}$ using only ten angular intervals. This confirms that for initially isotropic configurations, orientation subdivision is essential to accurately capture deformation-induced anisotropy.

In summary, for cases assuming uniformly distributed porosity changes, the spectral decomposition with orientation subdivision offers an excellent trade-off between computational cost and accuracy, being up to three orders of magnitude faster than full re-homogenization. The simple analytical update remains the fastest option but is limited to scenarios where constant hydrodynamic resistance is valid, delivering exact results with up to four orders of magnitude lower computation times.

### Full-scale liver perfusion

Modeling liver perfusion (Debbaut et al. 2014) is a relevant application for multi-compartment approaches (Rohan et al. 2018; Ebrahem et al. 2025). At the organ scale, the hepatic artery and the portal and hepatic veins with diameters of up to 1 cm provide blood supply and drainage. At the lobule scale, the blood is driven through sinusoids, small capillaries with a diameter of approximately 10 $\mu$m. The organ-scale vessels and the sinusoid microcirculation are connected by hierarchical vascular trees that consist of up to 20 levels of branches. The sinusoids are uniformly distributed throughout the entire liver volume, forming a three-dimensional network around rows of liver cells that are responsible for the metabolic liver functions.

**Fig. 17:**
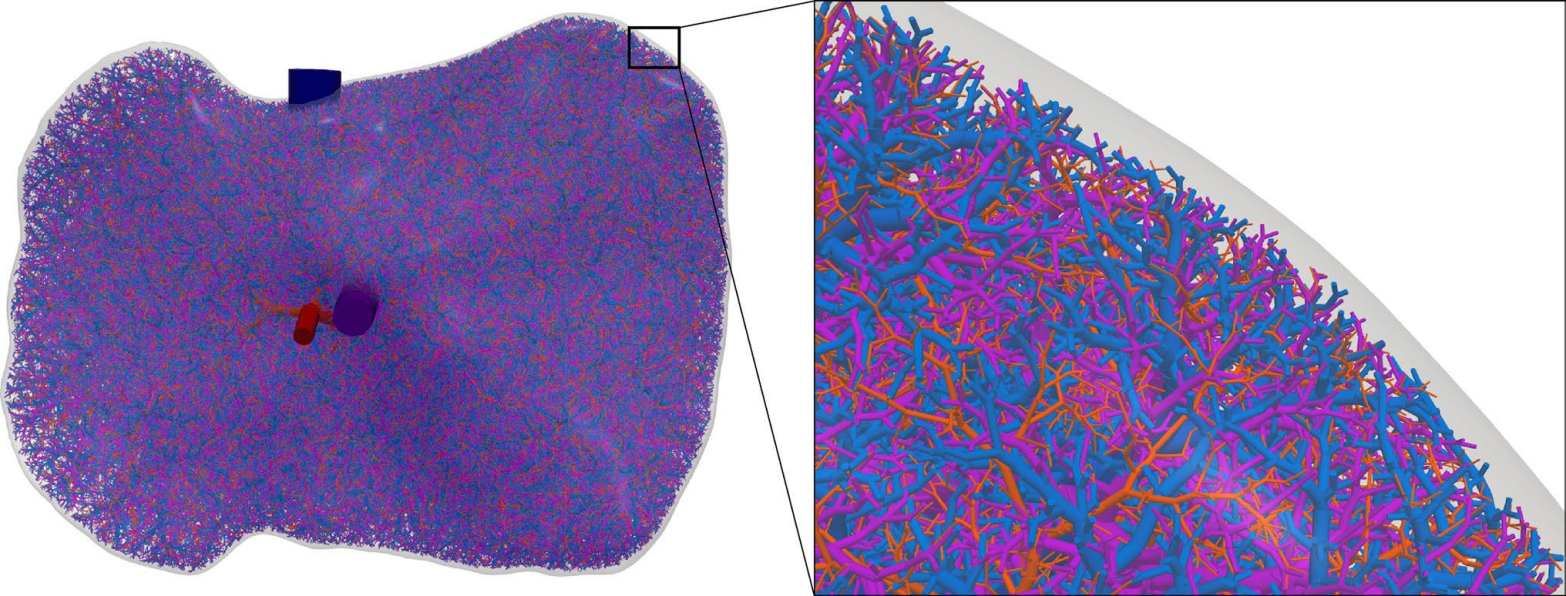
Synthetic vasculature for a patient-specific human liver: hepatic artery (red), portal vein (purple), hepatic vein (blue). We note that only the upper half of the vessel hierarchies are visualized

**Fig. 18:**
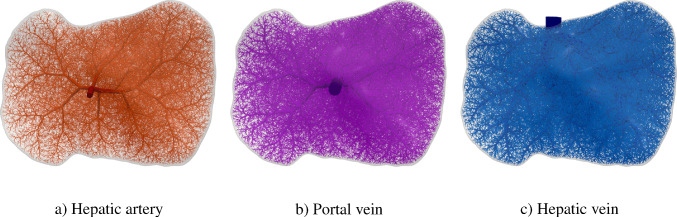
Separate plots of the three trees that constitute liver vasculature. We note that only the upper half of the vessel hierarchies are visualized

#### Patient-specific geometry, synthetic vascularization and model parameters

We use a patient-specific liver geometry obtained from CT scans provided in Bilic et al. (2023). For details on the segmentation and geometry preparation process, we refer to Ebrahem et al. (2025). We generate the vasculature of the liver synthetically via the framework described in Section 2, using the patient-specific liver domain and imposing the roots of the trees at the locations of the patient-specific roots. Figure 17 shows the patient-specific liver domain with the synthetically generated hepatic artery (ha), portal vein (pv), and hepatic vein (hv), each separately illustrated in Fig. 18. Each tree consists of more than 1.7 million vessels. The underlying parameters for synthetic tree generation are summarized in Table 4.

**Table 4:** Parameters for synthetic tree generation of the liver example

	$N_\text {vessel}$	$N_\text {term}$	$\Delta p$ [$\left[ {\frac{{{\mathrm{kg}}}}{{{\mathrm{mm}}{\mkern 1mu} {\mathrm{s}}}}} \right]$]	$\hat{Q}_\text {perf}$ [$\left[ {\frac{{{\mathrm{mm}}^{3} }}{{\mathrm{s}}}} \right]$]	$m_b$ [$\left[ {\frac{{\mu {\mathrm{W}}^{3} }}{{{\mathrm{mm}}^{3} }}} \right]$]	$\eta$ [$\left[ {\frac{{{\mathrm{kg}}}}{{{\mathrm{mm}}{\mkern 1mu} {\mathrm{s}}}}} \right]$]	$N_\text {resolved}$
Hepatic artery	1,781,564	890,782	1.176	4,000	0.6	$3.6 \times 10^{-6}$	18.681
Portal vein	1,767,920	883,960	0.217	16,000	0.6	$3.6 \times 10^{-6}$	100.968
Hepatic vein	1,756,428	878,214	0.045	20,000	0.6	$3.6 \times 10^{-6}$	234.590

We note that at the current resolution, the vascular trees remain one branching generation short of reaching the microcirculatory level. Consequently, the simulated pressure drop $\Delta p$ deviates from values reported in the literature and do not represent physiologically accurate values. Furthermore, typical physiological pressures are approximately up to 100 mmHg in the hepatic artery, 6–10 mmHg in the portal vein, and 1–5 mmHg in the hepatic vein, while the pressure in the microcirculation is typically 2–4 mmHg higher than in the hepatic vein (Debbaut 2013; Eipel et al. 2010).

We compartmentalize each of the lower hierarchies of the respective vascular tree into one compartment. We follow the above discussion regarding the RVE size and the vessel radius threshold. Vessels with a radius exceeding $r_{\textit{thresh}} = 0.2\,mm$ belong to the upper hierarchies that are kept resolved, while those with a radius of $r_{\textit{thresh}} = 0.2\,mm$ or smaller are assigned to the lower hierarchies. The number of resolved vessels $N_{\text {resolved}}$ for each tree is given in Tab. 4. We choose a radius of 5 mm for each spherical RVE to compute the homogenized material parameters in each finite element. For the standard deviation of the source distribution, we choose $\sigma = 3$ mm and set the isotropic and homogeneous permeability of the compartment microcirculation to $K_{\tiny {\text {micro}}} = k_{\tiny {\text {micro}}}/\eta = {1}/{180} \; \text {mm}^3~\text {s}~\text {kg}^{-1}$. For discretization, we use a finite element mesh of 870,626 four-noded tetrahedral elements.

#### Multi-compartment solution fields

**Fig. 19:**
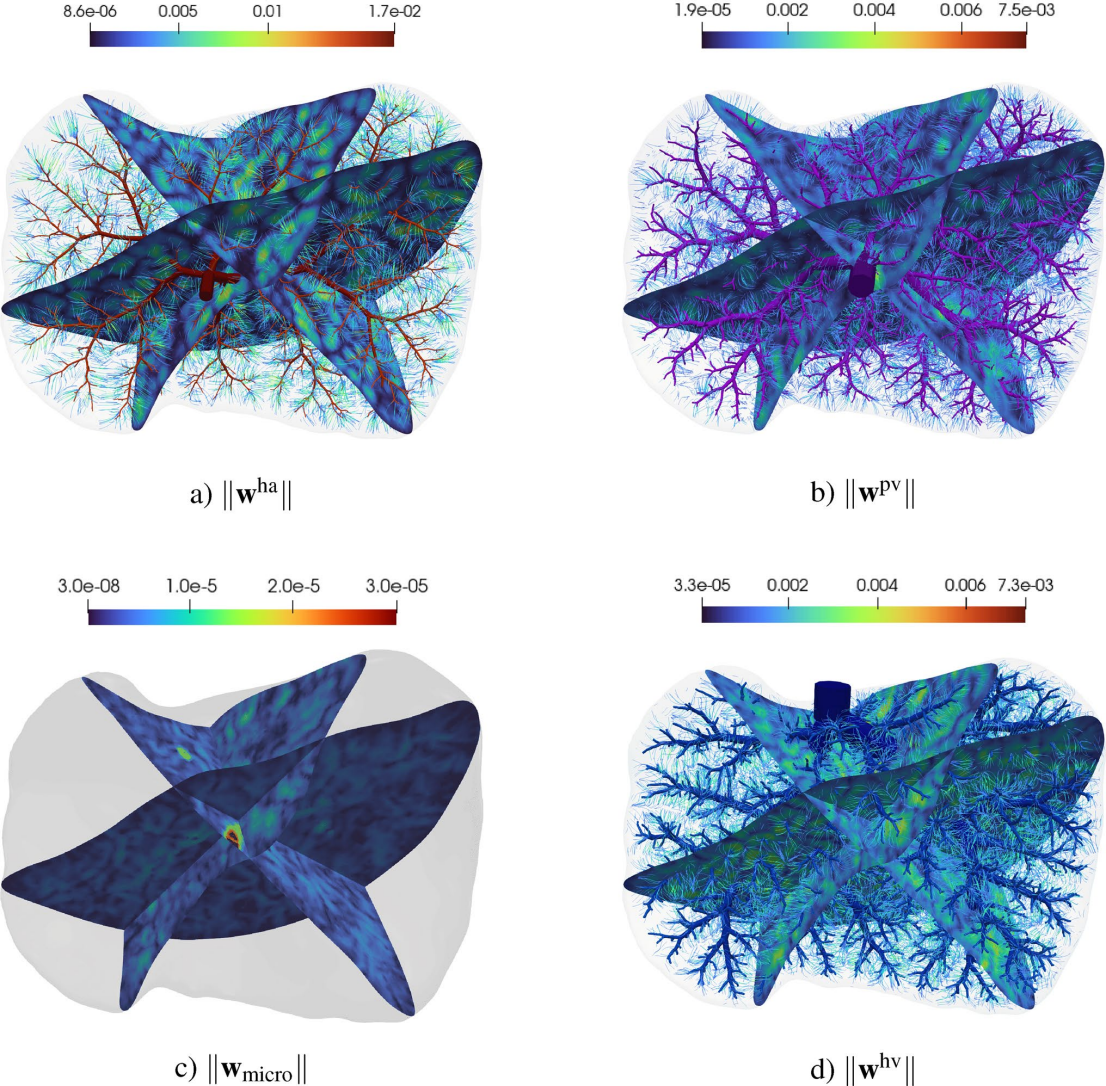
Magnitude of the filtration velocity fields for each compartment of the liver in [$\text {mm} \; \text {s}^{-1}$]. Flow directions are indicated by streamlines. Parts of the resolved upper hierarchies are shown

**Fig. 20:**
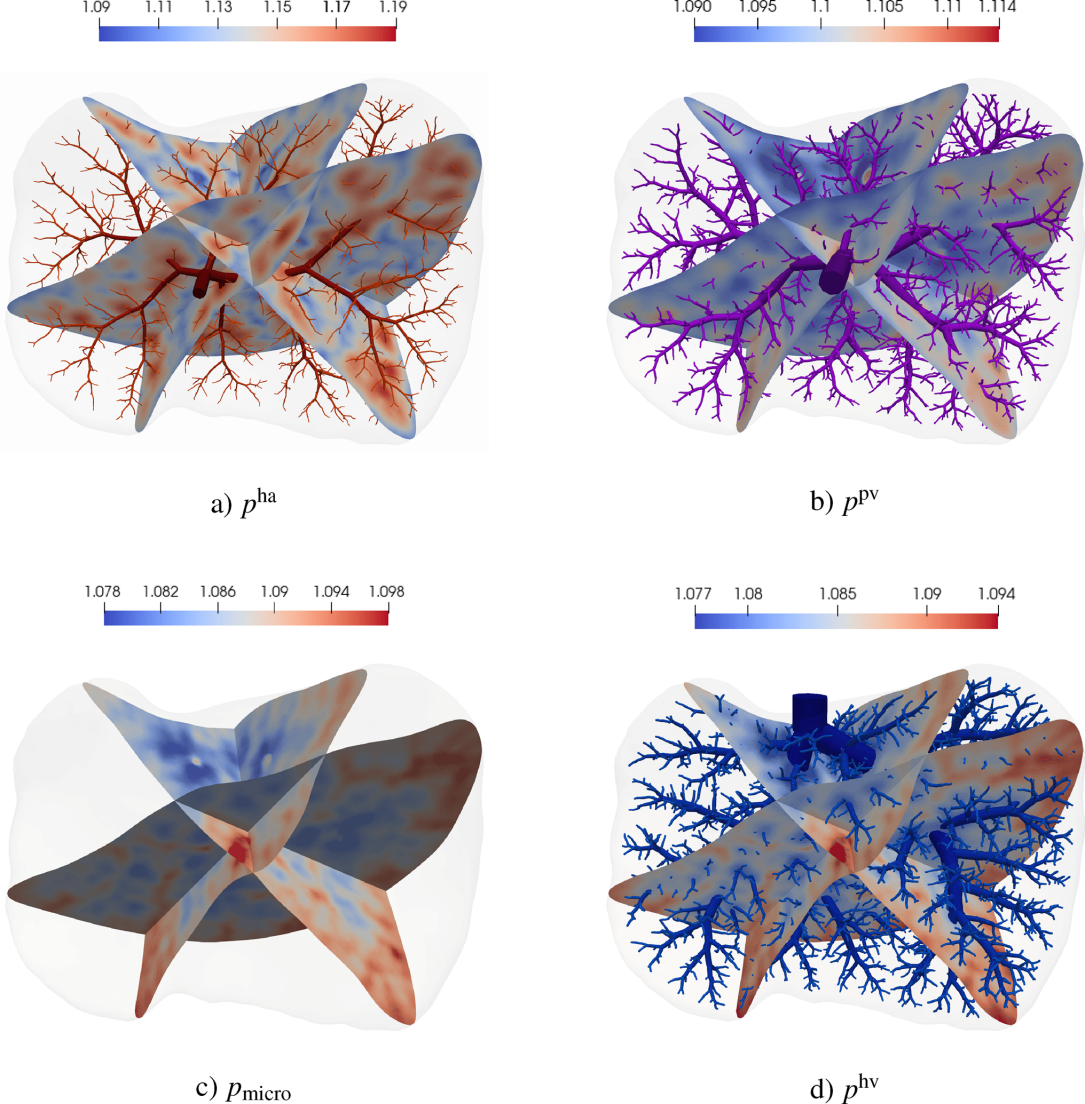
Pressure fields for each compartment of the liver in [$\text {kg} \; \text {mm}^{-1}~\text {s}^{-2}$]

**Fig. 21:**
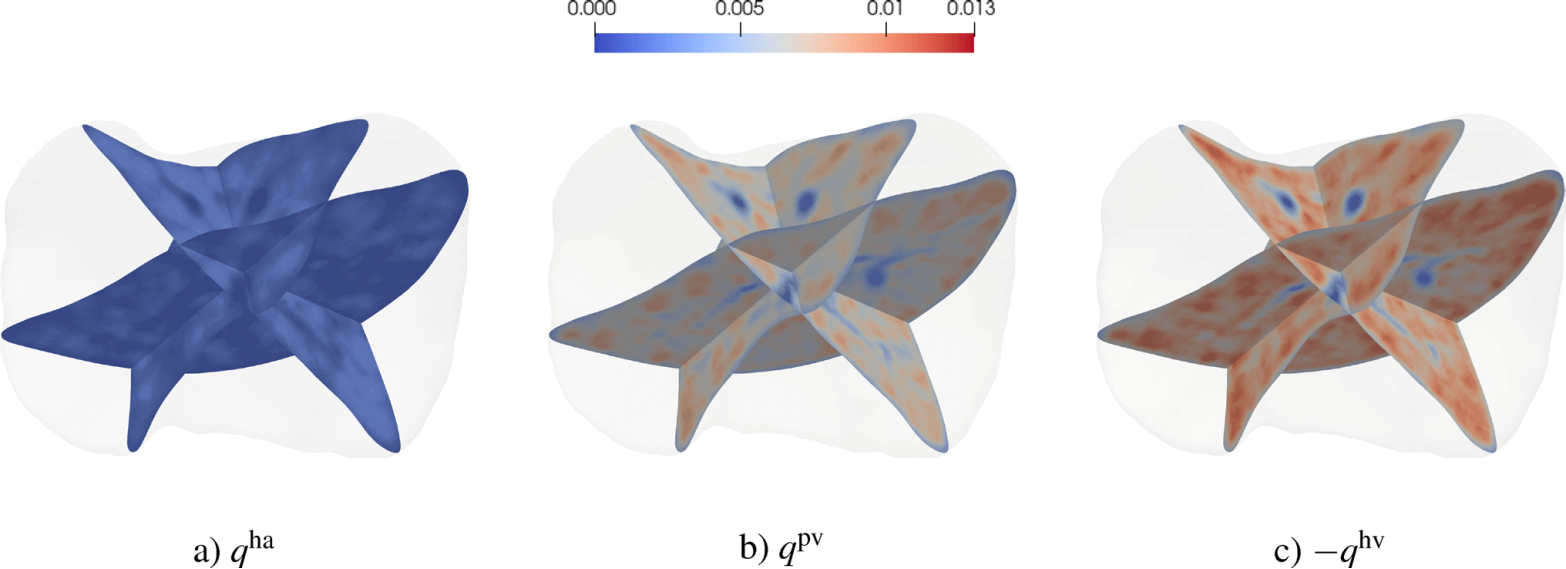
Intercompartmental flow rate densities in [$\text {s}^{-1}$] for the vascular tree compartments. The negative sign for compartment hv indicates that mass is drained from the microcirculation

**Fig. 22:**
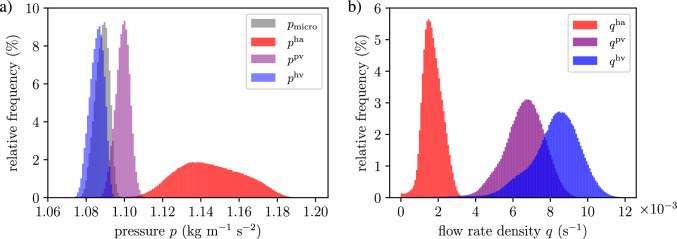
Variability of **a** the pressure in all liver compartments, and **b** the intercompartmental flow rate densities in and out of the microcirculation

Figure 19 illustrates the filtration velocity magnitudes for each compartment. We show slices overlaid with streamlines along with parts of the resolved upper hierarchy vasculature for ha, pv and hv. We observe that as in the 2D benchmark, the flow directions in all homogenized vascular tree compartments align with the dominant orientation of the larger homogenized vessels.

We can see that the velocity magnitudes in the microcirculation are approximately three orders of magnitude lower than in the vascular tree compartments. When vessel radii of upper hierarchy vessels exceed the RVE radius, only a few or no vessels are present within the RVE. To avoid numerical instabilities in these regions, we have assigned small finite values to the permeability. As a consequence, we accept that (artificial) velocity peaks occur at locations corresponding to the largest vessels in the upper hierarchies.

Figure 20 illustrates the pressure solutions for each compartment. For the vascular tree compartments. We include parts of the resolved upper hierarchy vasculature in the plots. We observe similar pressure patterns as in the 2D benchmark. The pressures exhibit pronounced pressure differences, following the structure of the respective resolved vasculature. In the compartments ha and pv, the pressure drops from regions with upper hierarchy vessels to regions without such vessels. This phenomenon becomes particularly clear in compartment ha, where the largest pressure drop occurs. In compartment hv, the pressure decreases further from regions of lower-hierarchy vessels to the upper hierarchy vessels and from there on to the root.

In contrast to the 2D benchmark, the microcirculatory pressure exhibits some spatial heterogeneity, bridging the pressure levels of the supplying compartments ha and pv and the draining compartment hv. Localized pressure peaks – particularly visible in the microcirculation and compartment hv – correspond to the location of larger vessels from the upper hierarchies, where the sparse presence of lower-hierarchy vessels and the absence of terminal branches lead to low permeabilities and vanishing perfusion coefficients.

Figure 21 displays the intercompartmental flow rate densities for the vascular tree compartments, representing mass supply from compartment ha and compartment pv to the microcirculation ($q^{\text {ha}}$, $q^{\text {pv}}$) and drainage into compartment hv ($q^{\text {hv}}$). We observe a generally uniform supply and drainage of the microcirculation, while the locations of the large tree segments of the upper hierarchies remain visible in regions with lower flow rate densities. Regions with minimal or zero intercompartmental flow rate density correspond to the same regions where velocity and pressure peaks occur. However, the intercompartmental flow rate density remains unaffected, as the intercompartmental perfusion coefficient vanishes in these regions.

The results indicate that our multi-compartment model with the chosen RVE size and the vessel radius threshold captures physiologically relevant hemodynamic behavior, demonstrating its ability to represent vascular trees with varying pressure drops, velocities, and microcirculatory perfusion within a unified modeling framework.

#### Variability analysis

To further support this conclusion, we construct histograms to compare the variability across the liver domain of the pressures in the different compartments and the flow rate densities into and out of the microcirculation. For simplicity, we sample data at the element midpoints of the tetrahedral mesh and weight the frequency by the element volume.

Figure 22 a illustrates the relative frequency of pressure values across different compartments. In compartment ha, we observe a broad range of pressure values, indicating a larger pressure drop. In contrast, compartments pv, hv, and the microcirculation exhibit similar pressure distributions and variabilities, suggesting comparable pressure drops, although their mean pressure values differ.

Figure 22 b displays the histogram of intercompartmental flow rate densities. The overall distribution patterns remain consistent across all vascular tree compartments. While compartments pv and hv exhibit similar variability, compartment ha shows lower variability and a lower mean value, indicating its reduced contribution to microcirculatory supply.

## Summary and conclusions

In this work, we presented a modeling framework that replaces parts of multiple synthetic vascular trees with interacting compartments governed by Darcy-type homogenized flow equations. We reviewed the governing equations based on mass balance principles to ensure a consistent representation of perfusion across different compartments and described a methodology to estimate permeability tensors and perfusion coefficients directly from synthetic vasculature.

Additionally, we developed two computationally efficient update mechanisms for deforming vasculature to capture permeability changes due to large deformations, which avoid computationally expensive re-homogenization in each iteration or time step: (1) a spectral decomposition approach balancing accuracy and efficiency while allowing incorporation of physiological conditions, and (2) a simple geometric update of the parameters, offering the fastest option but assuming constant hydrodynamic resistance. This assumption is based on significant simplifications, namely that remodeling and angiogenesis occur in organs with regenerative capacity over larger time scales. Therefore, rapid or transient dynamics cannot be captured under this assumption. This approach is not a general premise of the entire framework but represents the simplest possible physiological scenario to demonstrate how vessel-level assumptions can be incorporated into the permeability update formulation.

The model is sensitive to three key parameters: (1) the representative volume element (RVE) size, (2) the vessel radius threshold distinguishing resolved from homogenized vascular segments, and (3) the source distribution parameter controlling inflow spread. Due to physiological constraints, the scale separation between the organ level, the RVE, and fine-scale vascular features is limited to approximately one order of magnitude. Within these constraints, the RVE size and vessel radius threshold can vary only by a factor of two or three, with limited impact on model outcomes. An appropriately chosen RVE preserves the influence of larger vessels on permeability and perfusion, while an appropriately chosen source distribution parameter ensures that inflow remains consistent with the vascular structure and avoids over-localization.

We demonstrated the performance of the multi-compartment framework in hepatic perfusion simulations, showing that the homogenized model retains essential characteristics of the underlying vasculature, including distinct behaviors of the hepatic artery, portal vein, and hepatic vein. The model successfully captures physiologically relevant hemodynamic features such as pressure drops, velocities, and microcirculatory perfusion within a unified framework.

At the current state of development, there are several limitations of our approach that remain. While the homogenization scheme can incorporate non-Newtonian blood behavior such as the Fåhræus–Lindqvist effect in the initial homogenization process, the update relations currently assume constant viscosity during deformation. Capturing non-Newtonian effects dynamically poses challenges due to their strong nonlinearities, particularly in formulations similar to the spectral decomposition approach, where vessel properties are updated in an averaged sense. This simplification may be acceptable for larger vessel hierarchies but could introduce inaccuracies in capillary-scale flows.

Another limitation of the present study is the focus on steady-state simulations, neglecting transient dynamics and vessel compliance. Incorporating time-dependent mass balance equations and suitable discrete flow models for upper hierarchies could enable modeling dynamic phenomena, such as the diastolic flow patterns observed in coronary perfusion.

Additionally, the homogenized microcirculation compartment in our model, with its isotropic permeability, does not resolve detailed flow within the microcirculation and thus cannot capture distinct flow patterns at the macro- or mesoscopic scale. Our current synthetic generation method is limited to generate vasculature up to the pre-capillary level, and synthesizing realistic capillary beds would require further adaptations to the cost function. Extending synthetic vasculature into the microcirculation is essential for accurately capturing micro-flow characteristics and would enable validation of the homogenization approach against microvascular perfusion data. To fully assess the validity of this approach, rigorous comparative studies against anatomically realistic vascular networks remain necessary. While homogenization is a powerful tool, it is not the only viable method for modeling microperfusion, and direct comparisons with anatomical models are crucial to clarify its strengths and limitations.

Future work should address these limitations, investigate parameter sensitivity more comprehensively, and extend the model to incorporate tissue function, metabolism, and time-dependent effects. Experimental validation, particularly at the microcirculatory level, will be crucial for further refining the proposed framework and evaluating its predictive capabilities.

## Data Availability

No datasets were generated or analyzed during the current study.
